# Senktide blocks aberrant RTN3 interactome to retard memory decline and tau pathology in social isolated Alzheimer’s disease mice

**DOI:** 10.1093/procel/pwad056

**Published:** 2023-11-27

**Authors:** He-Zhou Huang, Wen-Qing Ai, Na Wei, Ling-Shuang Zhu, Zhi-Qiang Liu, Chao-Wen Zhou, Man-Fei Deng, Wen-Tao Zhang, Jia-Chen Zhang, Chun-Qing Yang, Ya-Zhuo Hu, Zhi-Tao Han, Hong-Hong Zhang, Jian-Jun Jia, Jing Wang, Fang-Fang Liu, Ke Li, Qi Xu, Mei Yuan, Hengye Man, Ziyuan Guo, Youming Lu, Kai Shu, Ling-Qiang Zhu, Dan Liu

**Affiliations:** Department of Pathophysiology, School of Basic Medicine, Tongji Medical College, Huazhong University of Science and Technology, Wuhan 430030, China; Department of Pathophysiology, School of Basic Medicine, Tongji Medical College, Huazhong University of Science and Technology, Wuhan 430030, China; Department of Pathology, The First Affiliated Hospital of Zhengzhou University, Zhengzhou 450002, China; Department of Pathology, School of Basic Medicine, Zhengzhou University, Zhengzhou 450002, China; Department of Pathophysiology, School of Basic Medicine, Tongji Medical College, Huazhong University of Science and Technology, Wuhan 430030, China; Department of Pathophysiology, School of Basic Medicine, Tongji Medical College, Huazhong University of Science and Technology, Wuhan 430030, China; Department of Pathophysiology, School of Basic Medicine, Tongji Medical College, Huazhong University of Science and Technology, Wuhan 430030, China; Department of Pathophysiology, School of Basic Medicine, Tongji Medical College, Huazhong University of Science and Technology, Wuhan 430030, China; The Second Affiliated Hospital, Department of Neurology, Hengyang Medical School, University of South China, Hengyang 421001, China; Department of Pathophysiology, School of Basic Medicine, Tongji Medical College, Huazhong University of Science and Technology, Wuhan 430030, China; Department of Pathophysiology, School of Basic Medicine, Tongji Medical College, Huazhong University of Science and Technology, Wuhan 430030, China; Beijing Key Laboratory of Aging and Geriatrics, National Clinical Research Center for Geriatric Disease, Institute of Geriatrics, Chinese PLA General Hospital and Chinese PLA Medical Academy, Beijing 100853, China; Beijing Key Laboratory of Aging and Geriatrics, National Clinical Research Center for Geriatric Disease, Institute of Geriatrics, Chinese PLA General Hospital and Chinese PLA Medical Academy, Beijing 100853, China; Beijing Key Laboratory of Aging and Geriatrics, National Clinical Research Center for Geriatric Disease, Institute of Geriatrics, Chinese PLA General Hospital and Chinese PLA Medical Academy, Beijing 100853, China; Beijing Key Laboratory of Aging and Geriatrics, National Clinical Research Center for Geriatric Disease, Institute of Geriatrics, Chinese PLA General Hospital and Chinese PLA Medical Academy, Beijing 100853, China; Department of Neurosurgery, Tongji Hospital, Tongji Medical College, Huazhong University of Science and Technology, Wuhan 430030, China; Department of Pathophysiology, School of Basic Medicine, Tongji Medical College, Huazhong University of Science and Technology, Wuhan 430030, China; Department of Pathophysiology, School of Basic Medicine, Tongji Medical College, Huazhong University of Science and Technology, Wuhan 430030, China; Department of Neurology, Union Hospital, Huazhong University of Science and Technology, Wuhan 430022, China; The Second Affiliated Hospital, Department of Neurology, Hengyang Medical School, University of South China, Hengyang 421001, China; Department of Biology, Boston University, Boston, MA 02215, USA; Center for Stem Cell and Organoid Medicine (CuSTOM), Division of Developmental Biology, Cincinnati Children’s Hospital Medical Center, Cincinnati, OH 45229, USA; Department of Pathophysiology, School of Basic Medicine, Tongji Medical College, Huazhong University of Science and Technology, Wuhan 430030, China; Department of Neurosurgery, Tongji Hospital, Tongji Medical College, Huazhong University of Science and Technology, Wuhan 430030, China; Department of Pathophysiology, School of Basic Medicine, Tongji Medical College, Huazhong University of Science and Technology, Wuhan 430030, China; Department of Medical Genetics, School of Basic Medicine, Tongji Medical College, Huazhong University of Science and Technology, Wuhan 430030, China

**Keywords:** Alzheimer’s disease, memory impairment, synaptic disorder, tau pathology

## Abstract

Sporadic or late-onset Alzheimer’s disease (LOAD) accounts for more than 95% of Alzheimer’s disease (AD) cases without any family history. Although genome-wide association studies have identified associated risk genes and loci for LOAD, numerous studies suggest that many adverse environmental factors, such as social isolation, are associated with an increased risk of dementia. However, the underlying mechanisms of social isolation in AD progression remain elusive. In the current study, we found that 7 days of social isolation could trigger pattern separation impairments and presynaptic abnormalities of the mossy fibre-CA3 circuit in AD mice. We also revealed that social isolation disrupted histone acetylation and resulted in the downregulation of 2 dentate gyrus (DG)-enriched miRNAs, which simultaneously target reticulon 3 (RTN3), an endoplasmic reticulum protein that aggregates in presynaptic regions to disturb the formation of functional mossy fibre boutons (MFBs) by recruiting multiple mitochondrial and vesicle-related proteins. Interestingly, the aggregation of RTN3 also recruits the PP2A B subunits to suppress PP2A activity and induce tau hyperphosphorylation, which, in turn, further elevates RTN3 and forms a vicious cycle. Finally, using an artificial intelligence-assisted molecular docking approach, we determined that senktide, a selective agonist of neurokinin3 receptors (NK3R), could reduce the binding of RTN3 with its partners. Moreover, application of senktide *in vivo* effectively restored DG circuit disorders in socially isolated AD mice. Taken together, our findings not only demonstrate the epigenetic regulatory mechanism underlying mossy fibre synaptic disorders orchestrated by social isolation and tau pathology but also reveal a novel potential therapeutic strategy for AD.

## Introduction

Alzheimer’s disease (AD) is the most common cause of dementia, which is characterized by a remarkably progressive decline of memory, language, thought, and problem-solving clinically (McKhann et al., 2011). AD can be divided into two categories: familial (also known as early-onset) and sporadic (also known as late-onset) AD. Familial AD is inherited in a Mendelian fashion and is mainly caused by mutations in three known genes (APP, PSEN1, and PSEN2). Late-onset AD (LOAD), comprising approximately 95% of cases, is much more complex because of the involvement of genetic, epigenetic, and multiple environmental components. Some risk factors, such as genetic mutations and ageing, cannot be changed, while a healthy lifestyle, including a Mediterranean diet, no smoking, regular physical exercise, and social activity may decrease the risk of cognitive decline and dementia (Livingston et al., 2020). According to meta-analysis data, diverse environmental factors over a person’s lifetime, such as low social support (Andel et al., 2012), current smoking (Hersi et al., 2017), alcohol use disorders (Schwarzinger et al., 2018), and sleep disturbances (Sindi et al., 2018) may contribute to the onset and development of AD cases (Barnes and Yaffe, 2011; Livingston et al., 2017). Of these, lifestyle factors, such as late-life social interaction, have well-characterized impacts on AD risk.

Loneliness and social isolation are recognized as serious public health risks that particularly affect the ageing society globally (Fakoya et al., 2020). Previous epidemiological studies have shown that loneliness and social isolation are associated with all-cause dementia, especially AD (Joyce et al., 2021; Sundstrom et al., 2020; Wilson et al., 2007). Social isolation, including low social participation, less frequent social contact, and more loneliness, led to cognitive inactivity and was significantly associated with incident dementia (Kuiper et al., 2015). In contrast, a recent study suggested a 4% reduction in dementia prevalence if social isolation were eliminated in later life (Livingston et al., 2017). Moreover, a 28-year follow-up of the Whitehall II cohort study suggested that more frequent contact confers higher cognitive reserve(Sommerlad et al., 2019). Although prolonged isolation stress during development exacerbates the onset of AD-related pathology and memory impairments in AD mouse model (Huang et al., 2015), which may be attributed to the effect of social isolation on the development, the effect of social isolation on the onset of AD and the mechanisms underlying the process without developmental factors remains unclear. Under the pandemic situation caused by COVID-19, social distancing (social isolation) has prominently increased in the ageing population (Hwang et al., 2020). Therefore, it is important to understand the impacts and detailed mechanisms of social isolation on the initiation of dementia.

Numerous studies have reported that loss of synaptic contacts in both the neocortex and the hippocampus is one of the key neuropathological findings in socially isolated (SI) individuals (Mumtaz et al., 2018), as well as in AD patients (Ferrero et al., 2018; Sze et al., 1997). The number of synapses is highly correlated with both Mini-Mental State Examination scores and delayed memory recall, suggesting the potential role of synaptic loss in memory impairments in the early stages of AD (Scheff et al., 2006). Among the widely distributed synaptic connections, the dentate gyrus (DG)-related circuits are of particular interest. The DG is increasingly recognized as an important structure involved in multiple memory tasks, including pattern separation (Leutgeb et al., 2007), pattern completion (Nakashiba et al., 2012), novelty detection (Fredes et al., 2021), and working memory (Sasaki et al., 2018). It is known that the DG receives excitatory inputs from the entorhinal cortex (EC) through the perforant pathway (PP) and recruits CA3 pyramidal cells to form the EC-DG-CA3 pathway during memory formation (Buzsaki, 2002; Hainmueller and Bartos, 2020). Previous studies have validated the critical role of DG granule cells (GCs) in memory encoding and retrieval (Kheirbek et al., 2013; Madronal et al., 2016; Tayler et al., 2013). Although it is known that the DG is resistant to the formation of plaques, tangles, and neuronal death until the late stages of AD, dysfunction of the DG-CA3 circuit has been implicated in synaptic disorders and memory impairments in the early stages of AD (Palmer and Good, 2011). For example, in an AD transgenic mouse model, the inhibitory circuits in the DG are reinforced, which leads to synaptic deficits (Palop et al., 2007). The loss of synaptic connections in the DG has also been reported in the inner and outer molecular layers of AD patients (Scheff and Price, 1998; Scheff et al., 1996). Loss of synaptic projection from the DG to the CA3 is highly correlated with cognitive impairments in AD (Llorens-Martin et al., 2014). Moreover, magnetic resonance imaging (MRI) studies have observed hyperactivity in the DG region, which was negatively correlated with cognitive performance in patients with amnestic mild cognitive impairment (aMCI), an MCI subtype at the highest risk of progression to AD (Yassa et al., 2010). Although the structural MRI data suggested that the grey matter volumes of hippocampus region were dramatically reduced in the socially isolated individuals (Shen et al., 2022), it remains unclear whether dysfunction of the DG-CA3 circuit is critical to the initiation of memory impairments by social isolation.

In this study, we first reported that 7 days of social isolation in the non-symptomatic AD mice but not WT mice triggered pattern separation impairments and DG-CA3 synaptic disorders by specifically inducing the presynaptic abnormalities. By screening the miRNAs extracted from the DG neurons collected by laser capture microdissection (LCM), we first reported that 2 of 40 DG-enriched miRNAs are specific downregulated (miRNAs downregulated in DG GCs of isolated AD mice, or miR-dDiAs) in the GCs of SI AD mice and 8 miRNAs are decreased in the non-symptomatic AD mice (preclinically downregulated miRNAs), as well as in the AD patients. We also found that epigenetic dysregulation (histone acetylation) plays an important role in the reduction of miR-dDiAs levels. A comprehensive target prediction analysis indicated that miR-dDiAs and preclinically downregulated miRNAs target transcripts of *Rtn3* and miR-dDiAs/*RTN3* signalling mediated the DG-CA3 synaptic dysfunction induced by social isolation. We further identified that aggregated RTN3 drives synapse loss by binding with a series of proteins involved in synaptic organization and mitochondrial functions. Moreover, by using molecular docking strategies and *in vitro* experiments, we screened senktide, previously known as a selective NK-3 tachykinin receptor agonist, which significantly blocked the physical interaction of RTN3 with its partners. Most importantly, the application of senktide effectively rescued the DG-CA3 synaptic disorders and prevented the pattern separation impairments induced by social isolation. These results uncover a novel epigenetic regulatory signalling pathway that mediates synaptic disorders in AD.

## Results

### Social isolation triggers pattern separation impairments in AD mice

**Figure 1.**
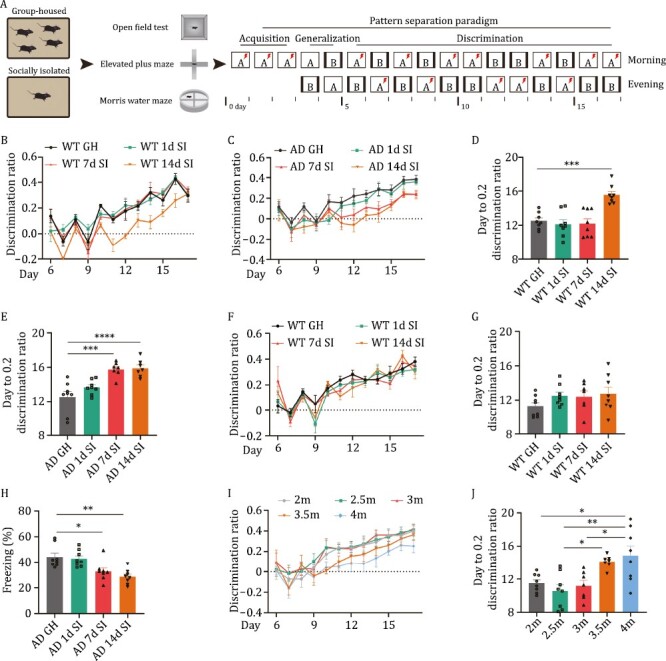
Social isolation triggers the pattern separation impairments in AD mice. (A) A diagram for the experimental procedures of the present study. WT or AD mice were group-housed (GH) or SI for 1, 7, or 14 days, and then subjected to an open field test, elevated plus maze test, Morris water maze test, and pattern separation tasks. (B and C) The discrimination ratio between two similar contexts [(*A* − *B*)/(*A* + *B*)] from day 6 to 17 in GH and SI WT mice (B) or AD mice (C). (D and E) The average days when the discrimination ratio reaches 0.2 in GH and different SI WT mice (D) or AD mice (E). ***P < 0.001, ****P < 0.0001. (F) The discrimination ratio between two similar contexts [(*C* − *D*)/(*C* + *D*)] from day 6 to 17 in GH and SI WT mice after one-month group housing. (G) The average days when the discrimination ratio reaches 0.2 in GH and different SI WT mice after one-month group housing. (H) The percent of freezing time in context C at day 3 of pattern separation task. *P < 0.05, **P < 0.01. (I and J) The discrimination ratio between two similar contexts [(*A* − *B*)/(*A* + *B*)] from day 6 to 17 (I) and the average days when the discrimination ratio reaches 0.2 (J) in AD mice at different ages. *P < 0.05, **P < 0.01. All results are represented as mean ± SEM. 1d, 1-day social isolation; 7d, 7-day social isolation; 14d, 14-day social isolation; GH, group-housed mice; SI, socially isolated mice; WT, wild-type mice; AD, Alzheimer’s disease model mice; 2m, 2-month-old mice; 2.5m, 2.5-month-old mice; 3m, 3-month-old mice; 3.5m, 3.5-month-old mice; 4m, 4-month-old mice.

To investigate the possible impact of social isolation on the cognitive function of AD mice, we subjected 2-month-old (non-symptomatic) 3× Tg-AD mice to 1-, 7-, and 14-day social isolation (Fig. 1A). After that, the mice were returned to group housing conditions. Compared with the group-housed mice, the isolated mice displayed no significant differences in exploratory, anxious, or locomotor behaviours, as indicated in the open field task and elevated plus maze task (Fig. S1A–E). The Morris water maze results suggested that the 14-day isolated AD mice but not WT mice exhibited slightly less spatial learning ability because they learned to find the hidden platform at day 4 but not day 3, as seen in the group-housed mice (Fig. S1F and S1G). No difference was detected in the memory retention and probe trial test (Fig. S1H–J), suggesting normal memory retention ability in the isolated group. We then employed the pattern separation paradigm to examine the ability to differentiate two similar contexts (Fig. 1A), which depends on the normal DG-CA3 circuit (Berron et al., 2016; McHugh et al., 2007). We found that neither the isolated nor group-housed mice showed any difference during the contextual fear acquisition and generalization stages (Fig. S1K–M). In the discrimination phase of the task from day 6 to day 17, the 7- and 14-day isolated AD mice while only 14-day isolated WT mice displayed a very significant deficit during the acquisition of the discrimination task, as shown by the discrimination ratio (Fig. 1B and 1C). We noticed that the discrimination ability began to be impaired from day 11 in 14-day isolated but day 12 in 7-day isolated AD mice during the discrimination task (Fig. 1C). To further evaluate the pattern separation, we performed a nonlinear regression with the individual discrimination ratio on different days and found that 7- and 14-day isolation significantly increased the average durations (days) when the discrimination ratio reaches 0.2 in AD mice (Fig. 1E), while only 14-day isolation in WT mice increased the average durations (Fig. 1D). We also found that the 1-day isolation exhibited anxiety-like behaviour, as shown by a minor decrement of time moving in the centre (Fig. S1C), which is consistent with a previous report (Zelikowsky et al., 2018). Interestingly, when the isolated WT mice and AD mice to novel two similar contexts C and D to examine the pattern separation after one-month group housing, the pattern separation deficits in 14-day WT mice were recovered (Figs. 1D, 1F, 1G, S1N and S1O) while the 7-day and 14-day isolated AD mice had developed to memory impairment (symptomatic), as shown as decreased freezing level in acquisition stage (Fig. 1H). As pattern separation deficits generally occur from 3.5 months in AD mice (Fig. 1I and 1J), these data suggested that social isolation promotes the initiation of pattern separation impairments in AD mice.

### Social isolation impairs the DG-CA3 circuit

**Figure 2.**
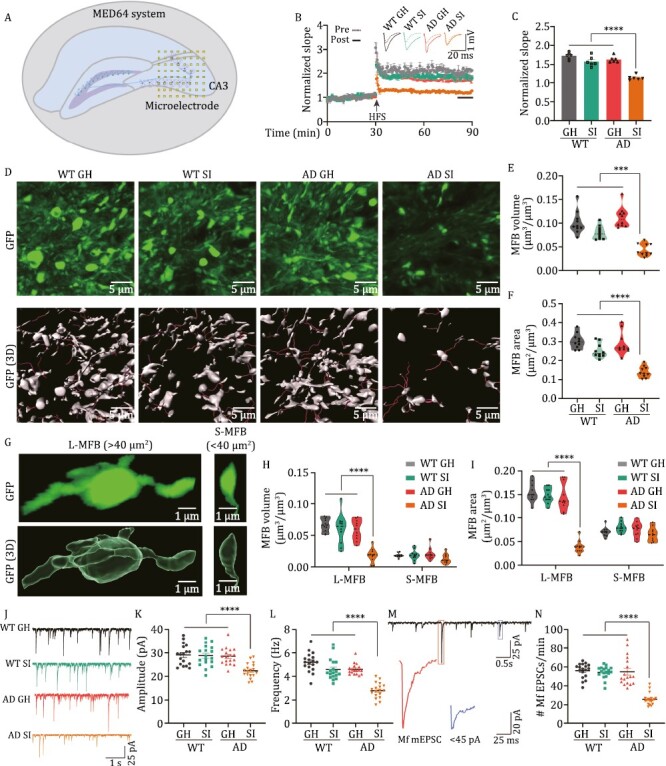
Social isolation impairs the DG-CA3 synapse in AD mice. (A) A diagram for LTP recording in the DG-CA3 synapse by MED64 system. The squares indicate the microelectrode array. (B) The normalized slope of fEPSP in different groups before (pre-) and after (post-) the high-frequency stimulation (HFS). Upper, the representative traces. Lower, the slopes of fEPSP. The arrow indicates the time for HFS. (C) Quantitative analysis of fEPSP slope at last 10 min as indicated by a black line in panel (B). ****P < 0.0001. (D) The representative fluorescent images (upper) and 3D reconstruction images (lower) of MFBs in the different groups. (E) Quantification of MFBs volume (MFBs volume μm^3^/volume of region of interest (ROI) μm^3^) in the different groups. ****P < 0.0001. (F) Quantification of MFBs surface area (MFBs surface area μm^2^/volume of ROI μm^3^) in the different groups. ***P < 0.001. (G) The representative fluorescent images (upper) and 3D reconstructive images (lower) for a single MFB. L-MFB: large MFBs with the area over 40 μm^2^; S-MFB: small MFBs with the area less than 40 μm^2^. (H and I) The volume of MFBs (H) and the surface area of MFBs (I) in the different groups. ****P < 0.0001. (J–L) The representative mEPSC traces (J), the amplitude (K), and the frequency (L) of mEPSC in the different groups. (M) The representative mEPSC traces and the illustration for the MF-based synaptic current. (N) The frequency of MF-based mEPSC in different groups. ****P < 0.0001. All results are represented as mean ± SEM.

It is known that both DG-CA3 projection and neurogenesis are implicated in pattern separation. We first examined adult neurogenesis in isolated AD mice. We found that only 14 days of isolation resulted in a decrease in neurogenesis in AD mice (Fig. S2). Therefore, the reduction in neurogenesis may not be responsible for the impaired pattern separation induced by the 7-day isolation in AD mice. We then evaluated the long-term potentiation (LTP) in the DG-CA3 circuit. We found that 7-day isolated AD mice (hereafter referred to as AD SI) led to a significant decrease in field excitatory postsynaptic potential (fEPSP) slopes (Fig. 2A–C), indicating impaired DG-CA3 synaptic plasticity. As the GCs in the DG directly project to CA3 pyramidal cells by forming a specific type of giant presynaptic structure, namely, MFBs (Monday et al., 2022), we then evaluated the morphological changes of MFBs in the stratum lucidum of CA3 region. By using confocal Z-stack imaging, we found that the surface areas and volumes of MFBs were dramatically reduced in the isolated AD mice but not in WT mice (Figs. 2D–F and S3A). We also described MFBs for each group and drew the density plot of the surface areas and volumes. We found that both curves resembled lognormal distributions, that is, long-tailed distributions with small numbers of larger areas/volumes and large numbers of smaller areas/volumes (Fig. S3B–E). As large MFBs (surface area > 40 μm^2^) mainly innervate the spiny excrescences of CA3 pyramidal neurons and hilar mossy cells, which are important for high synaptic efficacy and plasticity at DG-CA3 pyramidal neurone synapses (Rollenhagen and Lubke, 2010). We then re-analysed the MFBs according to their areas (Fig. 2G) and found that AD SI mice dramatically reduced the surface areas and volumes of the large MFBs. No significant change was found in the 7-day isolated WT mice (WT SI) (Fig. 2H and 2I). When we plotted the cumulative curves of MFBs area or volume and calculated the proportion of MFBs on the left side of the curves that constituted 80% of the total MFB area (or volume), we found that this proportion was increased following 7-day isolation of both AD and WT mice (Fig. S3F–I). These data strongly suggested that social isolation induced the abnormalities of MFBs, especially large MFBs, which are important structures in the DG-CA3 synapses.

Morphological alterations of the presynaptic component are always accompanied by electrophysiological changes (Hatada et al., 2000). To this end, we measured the mEPSCs in the CA3 pyramidal neurons. We found that the frequencies and amplitudes of mEPSCs in the CA3 neurons of isolated AD mice were significantly reduced (Fig. 2J–L). We further divided the mEPSCs according to the amplitude into two categories: larger than 45 pA (reflecting the MF-based synaptic projections to CA3 regions) and the remaining (reflecting the other synaptic inputs to CA3 regions, i.e. perforant path, associational/commissural (A/C) fibres) (Viana da Silva et al., 2019) (Fig. 2M). We found that the frequency of mEPSCs larger than 45 pA was much lower in isolated AD mice than in group-housed AD mice (Fig. 2N). However, the amplitude of mEPSCs larger than 45 pA (Fig. S3J) but not the remaining (Fig. S3L) were comparable in isolated and group-housed AD mice. Consistent with this finding, the Golgi staining results indicated that the isolated AD mice displayed a lower percentage of mature dendritic spines in the CA3 neurons (Fig. S3M and S3N). These data strongly suggested that chronic social isolation initiates the impairments of DG-CA3 circuit in AD mice.

### Social isolation disrupted the expression profiles of miRNAs in the DG GCs of AD mice

**Figure 3.**
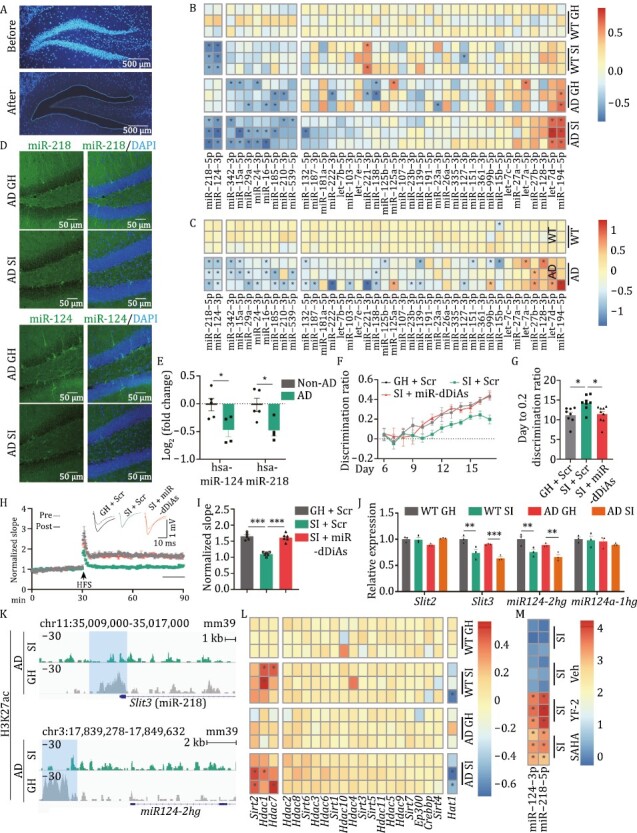
Social isolation epigenetically disrupts the expression of miRNA in the hippocampal DG area. (A) A representative image for the DG area before (upper) and after (lower) the laser capture microdissection. The nucleus was visualized by Hoechst 33342 (blue). (B) The heatmap for the expression profiles of 40 DG-enriched miRNAs in 7-day SI and group-housed (GH) WT and AD mice. The asterisk indicated Log2 (fold change of miRNA expression) > 0.5 or < −0.5. (C) The expression profiles of 40 DG-enriched miRNAs in the DG of 4-month AD and control mice. The asterisk indicated Log2 (fold change of miRNA expression) > 0.5 or < −0.5. (D) The representative FISH images for the miR-218 and miR-124 in 7 days socially isolated and group-housed AD mice. (E) The expression of has-miR-218 and has-miR-124 in the hippocampus of AD patients. *P < 0.05. (F) The discrimination ratio betweentwo similar contexts [(*A* − *B*)/(*A* + *B*)] from day 6 to 17 in miR-dDiAs agomirs or scramble treated group-housed or SI AD mice. (G) The average days when the discrimination ratio reaches 0.2 in differently treated AD mice. *P < 0.05. (H) The normalized slope of fEPSP in different groups before (pre-) and after (post-) the HFS. Upper, the representative traces. Lower, the slopes of fEPSP. The arrow indicates the time for HFS. (I) Quantitative analysis of fEPSP slope at last 10 min as indicated by a black line in panel G. ***P < 0.001. (J) The transcriptional levels of host genes for the miR-dDiAs in the DG of 7-day SI and GH WT and AD mice. **P < 0.01, ***P < 0.001. (K) The CUT&Tag results by using the antibody of H3K27ac. The blue shadow indicated the differentially binding peak. (L) The expression profiles of enzymes that are involved in the histone acetylation and deacetylation detected by qPCR. The asterisk indicated Log2 (fold change of gene expression) >0.4 or <−0.4. (M) The expression levels of miR-dDiAs in the DG neurons of isolated AD mice with the treatment of histone acetyltransferase activator YF-2 or pan-HDAC inhibitor SAHA or vehicle. The asterisk indicated Log2 (fold change of miRNA expression) >1.0 or <−1.0. All results are represented as mean ± SEM.

MicroRNAs (miRNAs) appear to be important modulators of posttranscriptional gene regulation in the nervous system and play critical roles in synaptic plasticity. We, therefore, dissociated the DG GCs in 7-day isolated and group-housed AD and WT mice by laser-captured microdissection (Fig. 3A). After verifying the quality of extracted RNA from the captured cells (Fig. S4A), we examined the expression levels of 40 miRNAs (Table S2) that were enriched in the DG region (Bot et al., 2013) by qPCR. We found that among those miRNAs, miR-218-5p, and miR-124-3p were decreased both in isolated AD mice and isolated WT mice (SI-induced downregulated miRNAs), 8 of them (miR-342-3p, miR-15a-5p, miR-29a-3p, miR-24-3p, miR-16-5p, miR-185-5p, miR-210-3p, and miR-539-5p) displayed downregulation both in isolated and group-housed AD mice (preclinically downregulated miRNAs) but more apparently in isolated AD mice (Figs. 3B, S4B and S4C), suggesting the socially isolated (SI) not only directly induce the downregulation of some miRNAs but also boost the dysregulation of preclinically downregulated miRNAs. We further examined those miRNAs in the DG GCs at 4 months (symptomatic) in 3× Tg-AD mice and confirmed the decrement of both SI-induced downregulated miRNAs and preclinically downregulated miRNAs (Fig. 3C). Fluorescence *in situ* hybridization (FISH) experiments further confirmed that the decreases in miR-218-5p and miR-124-3p were apparent in the DG GCs of isolated AD mice (Fig. 3D). In the hippocampal tissues of AD patients, hsa-miR-218-5p and hsa-miR-124-3p were also significantly decreased (Fig. 3E), which is consistent with the previous report (Lau et al., 2013). Combination with these data, we proposed that social isolation may specifically downregulate miR-218-5p and miR-124-3p in AD mice, and cooperate with preclinically downregulated miRNAs to trigger the impairments of DG-CA3 synapse. We, therefore, refer to the two downregulated miRNAs (miR-218-5p and miR-124-3p) as miRNAs downregulated in DG GCs of isolated AD mice (miR-dDiAs). To explore whether miR-dDiAs play important roles in mediating the DG-CA3 synaptic disorders induced by social isolation in AD, we infused miR-dDiAs agomirs into the DG area of AD mice at 2 months to elevate the expression of miR-dDiAs directly. One week later, these mice were subjected to social isolation for 7 days and then the pattern separation paradigm above was applied. We found that compared with the scramble-infected AD SI mice, the discrimination ratio was apparently restored in the isolated AD mice infected with miR-dDiAs agomirs (Figs. 3F, 3G, and S4D). Moreover, the MF-CA3 LTP (Fig. 3H and 3I), as well as the frequency of mEPSCs larger than 45 pA (Fig. S4E–H) were also restored by the miR-dDiAs agomirs. Interestingly, miR-dDiAs agomirs also enhanced adult hippocampal neurogenesis (Fig. S4I and S4J). Therefore, miR-dDiAs is critical to the DG-CA3 circuit impairments in social isolated AD mice.

### Histone deacetylation mediates the downregulation of miR-dDiAs

We then queried why miR-dDiAs are decreased in AD mice upon social isolation. We first examined the precursor of miR-dDiAs and found that they displayed synchronous reduction in the isolated mice (Fig. S4K), suggesting that the transcription of miR-dDiAs was suppressed. Consistent with this finding, the transcription of host genes for miR-dDiAs was also decreased (Fig. 3J). Recently, an increasing number of studies have paid attention to the critical role of enhancers in the regulation of gene transcription (Stadhouders et al., 2012). We identified the binding peaks of H3K27ac, H3K4me1, and H3K4me3 marks in the upstream genomic loci of the miR-dDiAs (Fig. S4L and S4M) (Zheng et al., 2019). Through H3K27ac Cleavage Under Targets and Tagmentation (CUT&Tag) analysis, we found lower histone acetylation levels at the enhancers of miR-dDiAs in 7-day SI AD mice (Fig. 3K). These data indicated an imbalance of histone deacetylases (HDACs) and histone acetyltransferases (HATs). We then performed a series of qPCRs (*Hdac1-11*, *Sirt1-7, Hat1, p300, CBP*) and found that *Sirt2*, *Hdac1,* and *Hdac7* increased and *Hat1* decreased (Fig. 3L) in the DG GCs of isolated AD mice. Application of the histone acetyltransferase activator YF-2 and the pan-HDAC inhibitor suberoylanilide hydroxamic acid (SAHA) by intraperitoneal injection restored the expression of miR-dDiAs (Fig. 3M). These data indicated that social isolation inhibited the expression of miR-dDiAs through altering histone acetylation at enhancers.

### RTN3 is posttranscriptionally upregulated by miR-dDiAs

**Figure 4.**
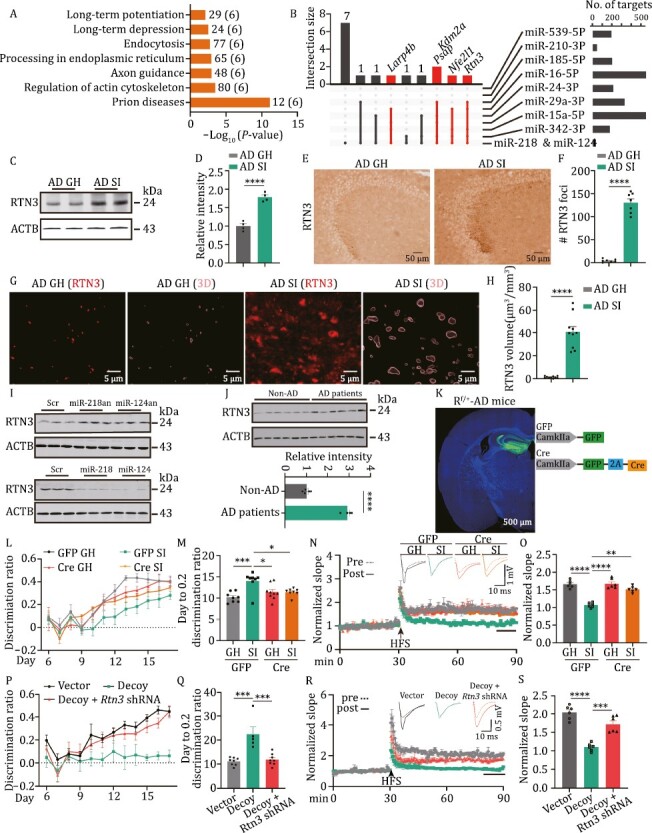
RTN3 is posttranscriptionally regulated by miR-dDiAs. (A) The KEGG pathway analysis for miR-dDiAs and preclinically downregulated miRNAs using Diana-mirPath (v.3). The numbers on the right of column indicated the number of genes targeted by miRNAs, the numbers in parentheses indicated the number of miRNAs. (B) Upset plot showing the targets of miRNAs. The red intersections indicated the genes targeted by miR-dDiAs and preclinically downregulated miRNAs. (C and D) The protein level of RTN3 was analysed by Western blot. (C) the representative blots; (D) the quantitative analysis. ****P < 0.0001. (E) The immunohistochemistry was used to evaluate the distribution of RTN3 in hippocampus of different groups. (F) The statistical analysis for the RTN3 foci in stratum lucidum of CA3 region of different groups. ****P < 0.0001. (G) The immunofluorescence images of RTN3 and the 3D reconstruction of RTN3 spots in stratum lucidum of GH or 7d SI AD mice. (H) The statistical analysis for the volume of RTN3 foci in stratum lucidum of different groups. ****P < 0.0001. (I) The HT22 cells were treated with the miR-218 and miR-124 antagomirs (an) (upper) or mimics (lower) with the scrambles as control and the lysis were collected for the Western blot analysis by using the RTN3 antibody. (J) The protein levels of RTN3 in the hippocampus of AD patients and non-AD control were analysed by Western blot. (upper) The representative blots; (lower) the quantitative analysis. ***P < 0.001. (K) The diagram for the virus injection in *Rtn3*^f/+^-AD (R^f/+^-AD) mice and the representative fluorescent image. (L) The discrimination ratio [(*A* − *B*)/(*A* + *B*)] from day 6 to 17 of pattern separation tasks of different groups. (M) The average days when the discrimination ratio value reaches 0.2 in different groups. *P < 0.05, ***P < 0.001. (N) The electrophysiological recording was performed in the DG-CA3 projection for LTP measurement. Upper, the representative traces; Lower, the normalized fEPSP slope. (O) Quantitative analysis of fEPSP slope was calculated for the last 10 min recording in (P). **P < 0.01, ****P < 0.0001. (P) The discrimination ratio [(*A* − *B*)/(*A* + *B*)] from day 6 to 17 of pattern separation tasks of different groups. (Q) The average days when the discrimination ratio value reaches 0.2 in different groups. ***P < 0.001. (R) The electrophysiological recording was performed in the DG-CA3 projection for LTP measurement. Upper, the representative traces; Lower, the normalized fEPSP slope. (S) Quantitative analysis of fEPSP slope was calculated for the last 10 min recording in (R). ***P < 0.001, ****P < 0.0001. All results are represented as mean ± SEM.

We then asked how miR-dDiAs contribute to DG-CA3 synaptic disorder triggered by social isolation. Bioinformation analysis with the Diana-mirPath (v.3) revealed the functional role of miR-dDiAs in axon guidance, endocytosis, and LTP (Fig. 4A and Table S3), which may be associated with DG-CA3 synaptic disorder. It is known that miRNAs regulate the translation of their targets at the posttranscriptional level by binding with the 3ʹ-UTR of targeted mRNAs (Lewis et al., 2005). We then employed the Encyclopedia of RNA Interactomes (ENCORI) database to analyse the mRNAs that may act downstream of miR-dDiAs and the preclinically downregulated miRNAs (Table S4) (Li et al., 2014). We found that miR-dDiAs and preclinically downregulated miRNAs potentially target prosaposin (*Psap*), nuclear factor erythroid-2, like-1 (*Nfe2l1*), RNA-binding protein la ribonucleoprotein 4B (*Larp4b*), lysine demethylase 2A (*Kdm2a*), and reticulon 3 (*Rtn3*) (Fig. 4B and Table S4). Among these targets, only PSAP and RTN3 exist in synaptic buttons (Wilhelm et al., 2014). Considering the elevation of PSAP was neuroprotective (Meyer et al., 2013) while RTN3 increment was implicated in the dystrophic neurititis (Hu et al., 2007) in AD, we, therefore, focus *Rtn3* as the target of miR-dDiAs to mediate the presynaptic dysfunction. The Western blot results indicated that RTN3 was upregulated in the hippocampus of isolated AD mice (Figs. 4C, 4D, S5A). Immunohistochemical data suggested that RTN3 was selectively increased in stratum lucidum, where the CA3 pyramidal neurone apical dendrites form synapses with DG granule cell axons (Fig. 4E and 4H). The nucleotide sequence analysis indicated that the 3ʹUTR of *Rtn3* contains multiple binding sites for miR-dDiAs (Fig. S5B). The luciferase assay showed that miR-218-5p and miR-124-3p directly bind to the wild-type 3ʹ-UTR of *Rtn3* mRNA but not the seed-region-mutated variants (Fig. S5C). Meanwhile, by application of the mimics and antagomirs of miR-218-5p and miR-124-3p, we found that the protein levels of RTN3 were decreased and increased, respectively (Figs. 4I, S5D and S5E). However, the mRNA levels of *Rtn3* were not changed (Fig. S5F and S5G), confirming the posttranscriptional regulation of RTN3 by miR-dDiAs. Moreover, in the hippocampal tissues of AD patients, RTN3 expression was significantly increased (Fig. 4J).

We then queried whether correcting the disturbed RTN3 signals could rescue the DG circuit impairments in isolated AD mice. To this end, we generated a hybrid mouse line by crossing *Rtn3* flox mice with AD mice, named R^f/+^-AD mice. Then, we injected AAV-packaged Cre virus into the DG area of R^f/+^-AD mice to suppress *Rtn3* expression *in vivo* (Figs. 4K and S6A–H). Then, these mice were subjected to social isolation. One week later, the pattern separation paradigm above was applied. We found that compared with the vector (GFP)-infected isolated R^f/+^-AD mice, the discrimination ratio was apparently restored in the isolated R^f/+^-AD mice infected with AAV-Cre virus (Fig. 4L and 4M). Moreover, the impairments in MF-CA3 LTP (Fig. 4N and 4O) as well as the frequencies and the amplitudes of mEPSC, especially the frequency of mEPSC larger than 45 pA (Fig. S6I–L) were reversed in *Rtn3* silenced mice. However, *Rtn3* knockdown did not improve adult hippocampal neurogenesis in isolated AD mice (Fig. S6M). Therefore, correcting the RTN3 signal is able to result in DG synaptic impairments in isolated AD mice.

To explore whether miR-dDiAs results in DG-related circuit disorders through RTN3 upregulation in isolated AD mice, we injected adeno-associated virus (AAV)-packaged miR decoy for miR-218 and miR-124 (AAV2/8-miR-dDiAs decoy, 1 × 10^12^ IU/μL, 1 μL) or mixed viruses of AAV-packaged decoy and AAV-packaged short hairpin RNA (shRNA) that specifically targets *Rtn3* (AAV2/8-miR-dDiAs decoy, 1 × 10^12^ IU/μL, 1 μL mixed with AAV2/8-*Rtn3* shRNA, 1 × 10^12^ IU/μL, 1 μL) or the control virus into the DG of group-housed c57 mice at 2 months (Fig. S7A–E). Two weeks later, we subjected the mice to the pattern separation task and found that the decoy-expressing mice, decoy + *Rtn3* shRNA-expressing mice and control mice accomplished the required tasks in the training stage from day 1 to day 3 and in the initial test stage from day 4 to day 5 (Fig. S7F and S7G). In the discrimination phase of the task from day 6 to day 17, control mice quickly learned to distinguish the similar chambers; however, the decoy-injected mice exhibited a very significant deficit during the acquisition of the discrimination task, and *Rtn3* knockdown partially rescued the impaired discrimination in the decoy-injected mice (Fig. 4P). Moreover, *Rtn3* silencing effectively reduced the days to reach a discrimination ratio of 0.2 (Fig. 4Q). The electrophysiological recording in MF-CA3 LTPs indicated that decoy-infected mice displayed a reduction in fEPSP slopes and that *Rtn3* shRNA partially rescued the reduction induced by the decoy (Fig. 4R and 4S). Thus, artificially manipulating the miR-dDiAs/RTN3 signals replicated the DG-CA3 circuit and memory impairments seen in isolated AD mice.

### miR-dDiAs/RTN3 signals impair the DG-CA3 circuit by disrupting presynaptic organization

**Figure 5.**
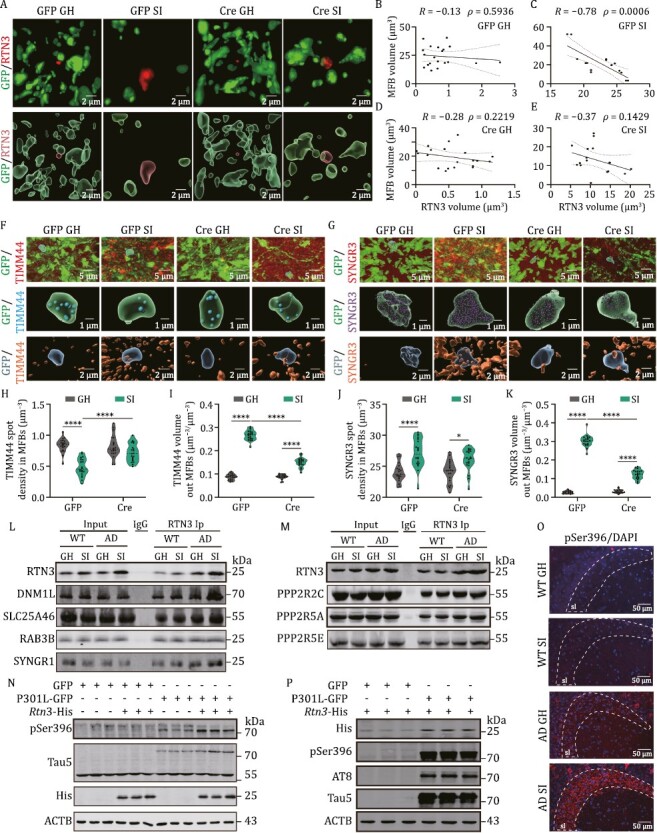
RTN3 impairs the presynaptic maturation in DG-CA3 circuit. (A) The immunofluorescence of RTN3 (red) with MFBs (GFP) in the stratum lucidum of CA3 region. Upper, representative Z-stack images; Lower, 3D reconstruction. (B–E) The correlation of RTN3 volumes with MFB volumes in (A). (F) The immunofluorescence of TIMM 44 with MFBs in the stratum lucidum of CA3 region. Upper, representative Z-stack images; Middle, 3D reconstruction of TIMM 44 inside of MFBs; Lower, 3D reconstruction of TIMM 44 outside of MFBs. (G) The immunofluorescence of SYNGR3 with MFBs in the stratum lucidum of the CA3 region. Upper, representative Z-stack images; Middle, 3D reconstruction of SYNGR3 inside of MFBs; Lower, 3D reconstruction of SYNGR3 outside of MFBs. (H–K) The quantitative analysis for TIMM44 (H and I) and SYNGR3 (J and K) foci inside or outside of the MFB. ****P < 0.0001. (L) Hippocampal lysis from the indicated mice were immunoprecipitated with RTN3 antibody and the pellets were subjected to the Western blot with the antibodies of DNM1L, SLC25A46, RAB3B, and SYNGR1. (M) Hippocampal lysis from the indicated mice was immunoprecipitated with RTN3 antibody and the pellets were subjected to the Western blot with the antibodies of PPP2R2C, PPP2R5A, and PPP2R5E in hippocampus. (N) The HT22 cells were transfected with 2 μg *Rtn3*-His and/or 2 μg P301L-EGFP or the vectors and the cell lysates were collected for Western blot. (O) The immunofluorescence of pSer396 in the stratum lucidum of CA3 region. (P) The HT22 cells were transfected with 0.5 μg *Rtn3*-His and/or 2 μg P301L-EGFP or the vectors and the cell lysates were collected for Western blot. All results are represented as mean ± SEM.

We then wanted to explore the downstream effectors of miR-dDiAs/RTN3 signals that disturb presynaptic assembly in MF-CA3 synapses. We noticed that RTN3 formed the aggresomes and the volumes of RTN3-positive aggresomes were negatively correlated with the surface areas and volumes of MFBs in SI AD mice (Fig. 5A–E). We then speculated that the aggregation of RTN3 abnormally recruited MFBs associated proteins. We dissociated the stratum lucidum of CA3 regions from isolated and group-housed AD mice and then subjected the homogenates to immunoprecipitation by using an antibody against RTN3. We selected three darker bands in the gel for isolated AD mice and sent them for the mass spectrum assay (Fig. S8A). We found that a total of 443 proteins were identified in these bands (fold change >1.5 compared to group-housed AD mice) (Table S5 and Fig. S8B–E). By using Gene Ontology analysis, we identified that these proteins were mainly enriched in receptor-mediated endocytosis and synaptic vesicle cycle pathways (Fig. S8F). Meanwhile, the gene set enrichment analysis suggested that these proteins were mostly associated with the mitochondria and synaptic vesicles (Fig. S8G), which are the core components in the MFBs. we proposed that the aggregation of RTN3 abnormally recruited these proteins and then interfered in the distribution of mitochondria and synaptic vesicles in the MFBs, which, in turn, inhibited the formation of MFBs. We, therefore, labelled the mitochondria and synaptic vesicles by immunostaining with antibodies against translocase of inner mitochondrial membrane 44 (TIMM44) and synaptogyrin 3 (SYNGR3), respectively. By employing 3D reconstruction with MFBs, we found that social isolation resulted in a dramatic reduction of mitochondria in the inside of MFBs compartment but a significant aggregation of mitochondria in the outside of MFBs. Consistent with previous reports (Shi et al., 2009a), the aggregation of synaptic vesicles can be detected both inside and outside of MFBs, indicating the abnormally swollen structure in the presynapse. However, the knockdown of RTN3 partially rescued the mislocalization of mitochondria and accumulation of synaptic vesicles out of the MFBs (Fig. 5F–K). Consistently, in isolated AD mice, RTN3 binds with more mitochondria and synaptic vesicles associated proteins, such as dynamin 1 like (DNM1L, functions in mitochondrial fission), solute carrier family 25, member 46 (SLC25A46, transmembrane protein of the mitochondrial outer membrane that controls mitochondrial organization and could regulate mitochondrial lipid homeostasis and thereby mitochondrial fission), RAS-related protein RAB3B (is anchored component of synaptic vesicle membrane and could regulate the synaptic vesicle cycle), and synaptogyrin-1 (SYNGR1, may play a role in synaptic-like microvesicle formation and/or maturation) (Fig. 5L).These data suggested that the aberrant RTN3 increment and aggregation disrupt the distribution of mitochondria and synaptic vesicles during MFBs dynamic reconfiguration in AD.

We then queried why social isolation-induced DG-CA3 synaptic dysfunction was irreversible by restoration of group housing in AD mice. We noticed that RTN3 could bind more PPP2R2C, PPP2R5A, and PPP2R5E, the important subunits of phosphatase 2A regulatory B, in the AD mice as shown in the mass spectrum and immunoprecipitation data (Fig. 5M and Table S5). The enhanced interaction of RTN3 with PP2A B subunits disrupted the formation of active PP2A complex (Fig. S9A–C), reduced the activity of PP2A (Fig. S9D), and finally led to the hyperphosphorylation of Tau (Figs. 5N, S9E and S9F). In line with it, the hyperphosphorylation of Tau was also obvious in the stratum lucidum of the CA3 region in isolated AD mice (Fig. 5O). While a previous study reported the level of Ser9-phosphorylated GSK-3β was significantly decreased in the isolated rats, we did not find the decreased level of Ser9-phosphorylated GSK-3β (Fig. S9G and S9H). Interestingly, overexpression of pathological Tau (P301L-Tau) could elevate the RTN3 level (Figs. 5P and S9I), suggesting RTN3 pathology and tau pathology may form a vicious cycle upon the social isolation treatment in AD mice.

### Senktide disturbs the binding of RTN3 with its partners and rescues the circuit disorder in isolated mice

**Figure 6.**
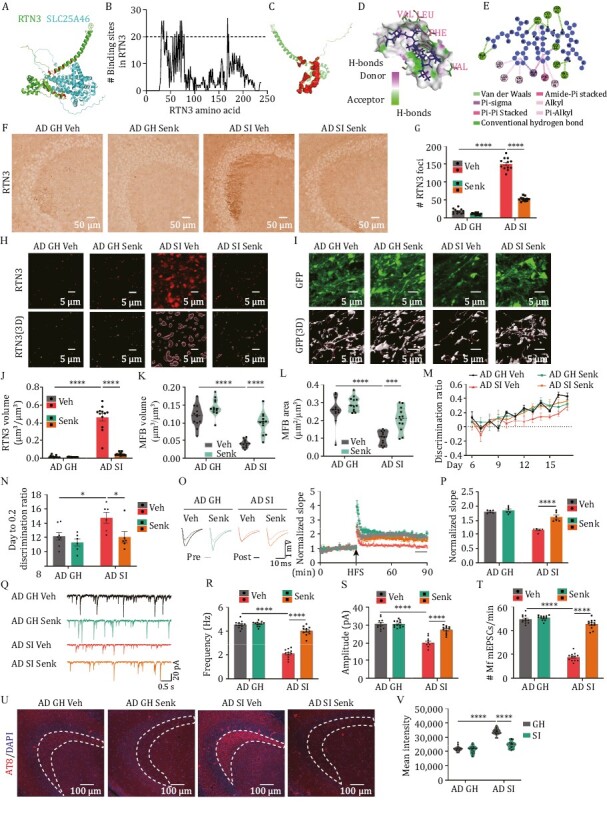
Senktide rescues the DG circuit impairments in isolated AD mice. (A) Structural model for RTN3 and SLC25A46 complex. The structure of RTN3 (green) and SLC25A46 (cyan) is from Alphafold protein structure database, and the complex of RTN3 and SLC25A46 were predicted from interEvDock3. The amino acids of RTN3 bound by SLC25A46 were shown as red. (B) 40 RTN3 partners associated with synaptic vesicles or mitochondria were chosen for the prediction of key binding sites in RTN3. The amino acid residues bound by more than 20 proteins were designated as key binding sites and selected as the binding pocket for molecule docking with small molecular compounds. (C) The structural models of the pocket (red) of RTN3 for molecular docking. (D) Hydrogen bond donor/acceptor of the binding site in RTN3 with senktide (blue). RTN3 is labelled with green while the amino acids interacting with senktide are labelled with magenta. The green dotted lines indicate the hydrogen bond between senktide and RTN3. (E) Docking interaction of senktide with RTN3. The dotted lines indicate interactions between senktide and RTN3. (F) The representative immunohistochemistry images using anti- RTN3 antibody in different groups. (G) The quantitative analysis of RTN3 foci in the stratum lucidum of the CA3 region. ****P < 0.0001. (H) The representative immunofluorescence images using anti-RTN3 antibody (upper) and the 3D reconstruction images (lower) in the stratum lucidum of CA3 region in the senktide (Senk) or vehicle (Veh) treated mice. (I) The representative images (upper) and 3D reconstruction images (lower) of MFBs in the stratum lucidum of the CA3 region. (J) The quantitative analysis of RTN3 volume in panel H. ****P < 0.0001. (K) The average volumes of MFBs in senktide or vehicle-treated mice. in the stratum lucidum of CA3 region. (L) The average surface area of MFBs in senktide or vehicle-treated mice. ***P < 0.001, ****P < 0.0001. (M) The discrimination ratio [(*A* − *B*)/(*A* + *B*)] from day 6 to 17 of pattern separation tasks of senktide or vehicle-treated mice. (N) The average days when the discrimination ratio reaches 0.2 in senktide or vehicle-treated mice. *P < 0.05. (O) The electrophysiological recording was performed in the DG-CA3 projection for LTP measurement in senktide or vehicle-treated mice. Left, the representative traces; Right, the normalized fEPSP slope. (P) Quantitative analysis of fEPSP slope at the last 10 minutes recording in (O). ****P < 0.0001. (Q–T) The representative mEPSCs traces (q), the mean frequencies (r), amplitudes (s), and the frequency of mEPSCs that are larger than 45 pA (t) in the senktide or vehicle-treated mice. ****P < 0.0001. (U) The immunofluorescence of AT8 (pS202, pT205) in the stratum lucidum of CA3 region. (V) Mean intensity of AT8 in the stratum lucidum (sl) of CA3 region. ****P < 0.0001. All results are represented as mean ± SEM.

Since the binding of RTN3 with its partners is important for the deficits of MFBs in isolated AD mice, we then tried to seek potential compounds to disrupt the binding of RTN3 with its partners associated with mitochondria and synaptic vesicles. To this end, we collected the structure of RTN3 and its binding partners from the AlphaFold Protein Structure Database (Varadi et al., 2022) and predicted their binding sites by using two online prediction tools (HDOCK (Yan et al., 2020) and interEvDock3 (Quignot et al., 2021)) (Fig. 6A). We found that 16 amino acid residues (S31, P32, G33, A34, P36, S42, W55, K59, F63, G66, T67, I70, L73, S74, F78, F168, and W170) in RTN3 were the top sites involved in the binding (Fig. 6B and Table S6). In the *in vitro* experiment, mutation of these amino acids partially disrupted the binding between RTN3 and its partners (Fig. S10A–E). We then set these 16 sites as a binding pocket and performed a molecular docking strategy to seek potential compounds that were possibly able to disrupt the binding of RTN3 with its partners by using a structure-based virtual screening approach (Fig. 6C). A total of 3,447 compounds from a list of drugs approved in major jurisdictions (Irwin et al., 2020) were analysed to yield 100,805 complexes and score each pose for physical complementarity to the RTN3 pocket. After analysing the top 200 poses, we selected 5 commercial compounds for further biological experiments (Table S7). Indeed, these 5 compounds ideally targeted the pocket and were predicted to form hydrogen bonds with A37, K41, S42, C43, G44, and V58 specifically (Figs. 6D, 6E and S10F–I). However, senktide was better at disrupting the binding of RTN3 with its partners *in vitro* (Fig. S10J–M). Considering that the brain–blood barrier is permeable to senktide (Stoessl et al., 1988; Zlomuzica et al., 2008), we then administered senktide (0.4 mg/kg/day, *s.c.*) to the 2-month-old isolated AD mice for 7 days during when the AD mice were SI (Fig. S10N). We found that senktide administration was able to reduce the aggregation of RTN3 in the MFBs (Fig. 6F–H and J) and disrupt the binding of RTN3 with its partners (Fig. S11A–J). Furthermore, senktide treatment not only attenuated the abnormalities in MFBs (Fig. 6I, 6K, and 6L) but also rescued the pattern separation impairments (Fig. 6M and 6N), LTP inhibition (Fig. 6O and 6P), reduced mEPSC frequencies (Fig. 6Q–T) and tau hyperphosphorylation (Figs. 6U, 6V and S11K) in the MF-CA3 circuit in the AD mice. Thus, blocking the binding of RTN3 with its partners effectively rescues DG-related circuit and memory impairments in isolated AD mice.

## Discussion

Social isolation has been widely applied to prevent and reduce the outbreak of COVID-19, while it also triggers a series of behavioural, emotional, and physical consequences, such as increasing the risk of cardiovascular, autoimmune, neurocognitive, and mental health problems, especially in older adults (Armitage and Nellums, 2020; Zhang et al., 2022). Given that COVID-19-infected individuals generally develop symptoms, including mild respiratory symptoms and fever, on an average of 5–6 days (mean 5–6 days, range 1–14 days) after infection (Liu et al., 2020) and 7-day and 14-day social isolation were widely used to avoid the spread of COVID-19, we choice 7-day and 14-day social isolation to evaluate the impact of this quarantine period on cognitive function. For AD patients, social isolation leads to more AD-like cognitive disorders rather than neuropathological changes (Azevedo et al., 2021; Wilson et al., 2007). Thus, it seems that social isolation could affect brain structure or plasticity independent of the progression of Aβ deposition. In our study, the Morris water maze reveals no difference between the mice while the pattern separation showed a very significant deficit. The Morris water maze and pattern separation are different behavioural paradigms that evaluate different abilities of cognitive function. Morris water maze is a versatile and pertinent tool for assessing spatial learning and memory, while pattern separation is used to estimate mnemonic discrimination between similar experiences. Pattern separation is based on intact learning and memory. A previous study reported that pattern separation was impaired in early AD mice but normal in the Morris water maze (Deng et al., 2020), which showed that pattern separation is a susceptible behaviour in the AD mice model, this is consistent with our finding. Previous studies have suggested that the disruption of hippocampal synaptic plasticity by social isolation results in behavioural abnormalities (Fone and Porkess, 2008). Many reports have indicated that adult neurogenesis in the DG, especially the survival and differentiation of newly divided cells, is dramatically reduced by social isolation in mice, rats, and guinea pigs (Ibi et al., 2008; Lu et al., 2003; Rizzi et al., 2007). In our study, we did observe a reduction in neurogenesis in 14-day but not 7-day isolated AD mice. These data suggested that the impaired pattern separation in 7-day isolated AD mice may not be due to the reduction of neurogenesis. GCs are the dominant cell types in the DG, and there is growing evidence indicating GC-related synaptic abnormalities in the early stages of AD. Impairment of mitochondrial Ca^2+^ uptake in GCs, which occurs at the age of 1–2 months in Tg2576 mice, contributes to mitochondrial dysfunction and DG-CA3 synaptic impairment (Lee et al., 2012). High-frequency priming stimulation (HPS)-induced LTP at EC-DG GC synapses is impaired in 3-month-old APP/PS1 female mice due to abnormal changes in the intrinsic excitability of GCs, resulting in dysfunctional information transfer from the EC to the DG (Jiang et al., 2021). Moreover, several studies have reported that enhanced tonic inhibition in dentate GCs strongly influences synaptic plasticity in AD mice, and pharmacological treatment to reduce tonic inhibition successfully rescues LTP impairment as well as memory deficits (Jo et al., 2014; Wu et al., 2014). In our study, we identified presynaptic loss, as indicated by abnormalities in the MFBs. However, the underlying mechanisms that mediate synaptic disorder in GCs induced by social isolation remain unclear.

miRNAs are small noncoding RNAs that target mRNAs for cleavage or translational repression, with a length of approximately 22 nt (Bartel, 2004; Beermann et al., 2016). It is known that miRNAs participate in many physiological and pathological processes, including development and innate immune, cardiovascular, and neurological diseases. In AD, the expression profiles of miRNAs in the blood, cerebrospinal fluid, and brain tissues have been well studied, and some of them are recognized as key regulators of senile plaques and neurofibrillary tangles. The critical role of miRNAs in the synaptic disorder of AD has also been illustrated (Kumar and Reddy, 2020). Some miRNAs, including miR-30b (Song et al., 2019), miR-34c (Hu et al., 2015b), miR-124 (Wang et al., 2018), and miR-132 (Xie et al., 2019), are involved in impaired synaptic plasticity in AD. In addition, quite a few miRNAs specifically located in the DG have been identified and play important roles in the synaptic plasticity of DG circuits under normal conditions (Ryan et al., 2017) and in disease (mental illness (Kohen et al., 2014), epilepsy (Zhang et al., 2017), etc.). Several miRNAs are known to regulate dentate granule cell development. For example, miR-132 is required for the maturation of newborn dentate GCs by targeting the GTPase-activating protein p250GAP, thereby mediating dendrite growth and spine formation (Magill et al., 2010). In turn, miR-132 has also been suggested to target BDNF and mediate its expression in the hippocampus (Ge et al., 2018; Xiang et al., 2015). Elimination of *Rncr3*, the main source of miR-124, results in dentate granule cell axonal mis-sprouting rather than normal neurogenesis, suggesting that miR-124 is essential for neuronal maturation and maintenance (Sanuki et al., 2011). Similarly, an *in vitro* luciferase assay proved that BDNF is directly targeted by miR-124, and a miR-124 antagomir significantly mitigates the depression-induced reduction in BDNF levels in the rat hippocampus (Yang et al., 2020). To date, however, there has been little discussion about how miRNAs exacerbate memory deficits through GC-related synaptic plasticity as part of a suite of social-isolation-related behavioural abnormalities in AD. Therefore, revealing the critical roles of specific miRNAs and their downstream molecules in granule cell synaptic dysfunction might be a promising source of therapeutic targets for AD. In this study, DG-enriched miRNAs (miR-dDiAs) were identified to be dysregulated in SI AD mice. Moreover, the disrupted miR-dDiAs together with preclinically downregulated miRNAs co-regulate the expression of RTN3 posttranscriptionally and then impair MF-CA3 synaptic plasticity at the presynaptic level. Application of the miR-dDiAs agomirs was able to rescue the MF-CA3 synaptic disorder in SI AD mice. As the deregulation of miR-218 and miR-124, the two miR-dDiAs, has been implicated in the pathogenesis of AD and the dysfunction of synaptic plasticity (Lu et al., 2021; Wang et al., 2018), our data further illustrated the coregulation of environmental changes with epigenetic factors in AD.

We also identified that RTN3, a member of the reticulon family of proteins that accumulated in the dystrophic neuritis of AD brains (Hu et al., 2007; Shi et al., 2009a), was specifically elevated in the MFBs of DG projections. The upregulation of RTN3 in MFBs impairs synaptic transmission and synaptic plasticity in the DG-CA3 circuit in SI AD mice. Although many studies have shown that RTN3 inhibits the APP process by directly interacting with β-secretase (BACE1) (He et al., 2004; Shi et al., 2009b), another important function of RTN3 in the formation of RTN3-immunoreactive dystrophic neurites (RIDNs) was emphasized in the pathogenesis of AD (Shi et al., 2009a). In mice that overexpress human *Rtn3* cDNA, a reduction in LTP followed by abundant RIDNs in the hippocampus can be detected (Hu et al., 2007). Moreover, loss of dendritic arborization and dendritic spines, as well as impaired anterograde axonal transport, was also found in Tg-*Rtn3* mice (Shi et al., 2009a). These lines of evidence strongly suggest that RTN3 acts as a negative regulator in synapses. Here, we found that *Rtn3* could act as a key downstream effector of miR-dDiAs to mediate presynaptic disorder. Among all of the deregulated miRNAs in DG GCs, *RTN3* can be posttranscriptionally modulated by 6 miRNAs (miR-dDiAs and 4 preclinically downregulated miRNAs). Interestingly, RTN3 was elevated in the MFBs, a presynaptic structure in the CA3 region. Manipulating the miR-dDiAs/RTN3 signal replicated the presynaptic disorders, including the reduction in mEPSC frequency and aberrant MFBs morphology, as seen in isolated AD mice. In line with our data, RTN3 was colocalized with synaptophysin (a presynaptic marker) in the tubulovesicular structures and varicosities of developing axons (Kumamaru et al., 2004). Furthermore, RTN3 was recognized as an endoplasmic reticulum (ER) marker, and the upregulation of RTN3 can also be found in response to ER stress (Grumati et al., 2017), which was implicated in presynaptic calcium imbalance (Ozturk et al., 2020). Therefore, our data extend the critical role of RTN3 in presynaptic disorders in AD.

We finally reported that the application of senktide is able to disrupt the interaction between RTN3 and its numerous partners and rescue the impairment in the DG circuits and pattern separation in isolated AD mice. It is known that senktide is a selective agonist of neurokinin3 receptors (NK3R) and has been suggested to excite a subpopulation of dopamine-sensitive neurons to enhance dopaminergic function (Keegan et al., 1992) and induce locomotor activity (Nordquist et al., 2008). Administration of senktide to aged animals preserves memory for object location in the object-place recognition task and for the location of a hidden platform in the Morris water maze test (de Souza Silva et al., 2013). Moreover, the application of senktide was effective in improving cognitive functions in rats with AD pathology. The protective effects of senktide on AD rats may rely on the enhanced cholinergic system (Koca et al., 2021). In contrast, knockout of NK3R in mice led to a series of cognitive deficits, as seen in the passive avoidance test, conditioned avoidance response and Morris water maze (Siuciak et al., 2007). Notably, we screened senktide by targeting the interaction between RTN3 and its partners in isolated AD mice. We also noticed that senktide did not reduce the overload of RTN3 (Fig. S10) but effectively restored the DG circuit and pattern separation, which further verified the important role of the abnormal interactomes of RTN3 in AD.

**Figure 7.**
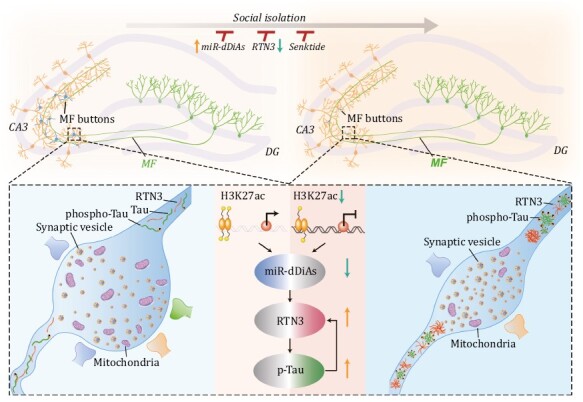
Proposed working model of social isolation triggers the DG circuit disorders in AD. In normal conditions (left), miR-dDiAs were epigenetically regulated by H3K27ac marks in the promoter regions and simultaneously regulates the proper expression of RTN3, which was essential for normal DG circuit and functions. Social isolation, specifically downregulates the H3K27ac marks and the expression of miR-dDiAs, results in the overexpression and aggregation of RTN3 in the presynaptic fraction, which, in turn, disturb the formation of mature MFBs by recruiting multiple mitochondrial and vesicle-related proteins, then leads to the AD-like disorders of DG synapses and pattern separation. RTN3 elevation also led to the tau hyperphosphorylation, which, in turn, promoted the RTN3 aggregation. Rebuilding the miR-dDiAs/RTN3 signals by genetic approaches or blocking the binding of RTN3 with its partners rescued those abnormalities.

Taken together, our findings not only reveal novel underlying mechanisms of epigenetic regulation in the impaired DG circuit caused by social isolation in AD but also provide novel therapeutic targets and drugs for the treatment of early-stage AD (Fig. 7).

## Methods

### Animals

Male 3× Tg-AD mice were purchased from the Jackson Laboratory (Bar Harbor, ME, NO.034830). Adult male C57BL/6 mice were purchased from the National Resource Center of Model mice (Nanjing, China). *Rtn3*^flox/flox^ mice were generated from Gempharmatech Co., Ltd (Jiangsu, China). To obtain *Rtn3*^flox/+^-AD mice, *Rtn3*^flox/flox^ mice were crossed with 3× Tg-AD mice. To confirm knockin of loxp sequence, genome DNA from the tail was amplified using the primers 5ʹ-GCTGTTGACTCTGAACCTTCCATG-3ʹ and 5ʹ-CAACAACAAAGCAGTCCAGCTC-3ʹ. All these mice and their littermates were bred in the Experimental Animal Central of Tongji Medical College, Huazhong University of Science and Technology, and housed under a 12-h light/dark cycle in a temperature (22–24°C) and humidity (40%–60%) controlled room with food and water ad libitum. This study was approved by the Institutional Animal Care and Use Committee of the Huazhong University of Science and Technology. Social isolation was performed as previously described (Zelikowsky et al., 2018). Mice were housed in isolation for 24 h, 7 days, and 14 days (SI, one animal per cage), or in group of four mice (Group-housed). All cage conditions remained identical to isolated mice compared to group-housed mice.

### Human hippocampal samples

Postmortem hippocampal tissues from six patients with AD and six nondementia age-matched control subjects based on neuropathological diagnosis were provided by the Tissue Bank of Institute of Geriatrics, Chinese PLA General Hospital and Chinese PLA Medical Academy. detailed patient information is listed in Table S1. Informed consents were obtained from all the subjects. The present study was approved by the ethics committee of Tongji Medical College (Wuhan, China).

### Cells

Human HEK293 cells were bought from the American Type Culture Collection bank (Manassas, VA, USA). Mouse HT22 cells were bought from the Millipore sigma (cat#SCC129). The cells were cultured in DMEM (Pricella, cat#PM150210) with 10% FBS (MIKX Co. Ltd, cat#MK1124-500).

### Laser capture microdissection

Frozen tissue was sectioned into 12-μm slices and placed on an MMI Membrane Slide (Molecular Machines & Industries, 50103). Tissue sections were incubated with Hoechest 33342 (Sigma Aldrich, B2261) on ice for 10 min, washed in RNase-Free PBS, and dried. Afterward, the MMI Membrane Slide is inverted and placed onto a glass slide for protection against contamination. GCs in the DG were identified and laser microdissected using MMI CellCut. The isolated cells were collected by MMI Isolation Caps (Molecular Machines & Industries, 50202) for transcriptional analysis.

### Plasmids and viruses

miR-218-5p, miR-124-3p mimics/agomirs/antagomirs, and the scrambled control were purchased from RiboBio (Guangzhou, China). Wild-type and mutant 3ʹUTR of *Rtn3* were amplified and cloned into psiCHECK-2 (Promega, Madison, WI). The coding sequences for the mRNA of *Rtn3* (NM_053076), *Slc25a46* (NM_026165), Syngr1 (NM_207708), *Rab3b* (NM_023537), and *Dnm1l* (NM_152816) were cloned into pcDNA3.1(+)-6× his or pcDNA3.1(+)-3× Flag. The mutant *Rtn3* plasmids were constructed using Mut Express MultiS Fast Mutagenesis kit V2 (Vazyme, Nanjing, China). AAVs for miR-218 & miR-124 decoy and *Rtn3* short hairpin RNA were purchased from OBio technology (Shanghai, China). The target sequence of *Rtn3* shRNA was 5ʹ-AGACCATAGUGGGGUGGAGCU-3ʹ.

### Morris water maze

The Morris water maze was performed as previously described (Zheng et al., 2021). Briefly, marks with different shapes were pasted around the swimming pool. Mice were trained to find the hidden platform three sessions a day for six consecutive days, starting from three different quadrants to the platform. Each session lasted for 1 min. If the mice found the platform less than 1 min, it will stay on the platform for 15 s. If the mice could not find the platform within 1 min, the operator will guide the mice to find the platform and stay for another 15 s. On the seventh day, mice were placed in the opposite quadrant of the platform to detect the latency to the platform within 1 min. After 48 h, a probe trial without the platform was used to detect the memory retention.

### Contextual fear conditioning, generalization, and discrimination

Contextual fear conditioning, generalization, and discrimination were performed as previously described with modifications (Sahay et al., 2011). Before the testing, mice were allowed to habituate for 1 h in the testing room. In the fear memory acquisition phase (from day 1 to day 3), Mice behaviours were recorded by digital video cameras mounted above the conditioning chamber. The chamber had a plexiglass front, three plastic walls, and a stainless-steel grid as a floor connected via a cable harness to a shock generator, and measured 30 × 25 × 21 cm. Beneath the chamber, a solution of 1% acetic acid was placed to provide an olfactory cue (context A). Once placed in the chamber, mice were allowed to freely explore for 185 s, then received a single, unsignalled 0.75 mA footshock (2 s in duration). After the shock, the mice remained in the chamber for 1 min, and then, they were returned to their home cages. The chamber was cleaned with 70% ethanol before the next test. Freezing was assessed for the first 185 s. In the generalization phase (day 4 and day 5), mice were placed into context A for 5min in the morning and placed into the context B with a mild mint scent as the olfactory cue and two plastic inserts covering the side walls in the evening, and freezing was recorded for 5 min on both contexts. On day 5, the order of the two contexts was reversed. For the discrimination phase (from day 6 to day 17), mice were placed into the context A with a single 2-s footshock of 0.75 mA after 185 s exploration and remained for 1 min after footshock and placed into the context B for 247 s daily. The order of exposure on each day was BAABBAABBABAABABBAABBABA. The first 3 min of freezing levels in both contexts were recorded for computing the discrimination ratio: (Freezing_context A_ — Freezing_contextB_)/(Freezing_context A_ + Freezing_contextB_). The curves of discrimination ratio with different days were fitted using nonlinear regression to second-order polynomial (quadratic). Contextual fear conditioning, generalization, and discrimination in contexts C and D were performed similarly as in contexts A and B except that the room set contexts C and D were different from the room set contexts A and B, and in contexts C, the six God toilet water was used to provide an olfactory cue, while in the context D, a solution of 1% curry powder was used. The walls of context C and context D were covered by polyvinyl chloride wallpapers with different thicknesses and textures.

### Dual luciferase report assay

Dual luciferase report assay was performed as previously described (Wang et al., 2018). The 200 bp sequence in 3ʹUTR of *Rtn3* flanked in the sequence targeting to miR-218 and miR-124 was amplified by PCR and was inserted into psiCHECK-2. The plasmids were transfected into HEK293 WT cells and cells were harvested and lysed for testing firefly and renilla luciferase activities using the dual luciferase reporter assay kit (Promega) according to the manufacturer’s protocol.

### Electrophysiological recording

Electrophysiological recording was performed as previously described (Hu et al., 2015a). Mice were decapitated after anaesthesia and the brains were immediately immersed into ice-cold artificial cerebrospinal fluid (125 mmol/L NaCl, 2.0 mmol/L KCl, 2.5 mmol/L CaCl_2_, 1.2 mmol/L MgSO_4_, 1.2 mmol/L KH_2_PO_4_, 26 mmol/L NaHCO_3_, and 11 mmol/L glucose), which was continuously bubbled with 95% O_2_ and 5% CO_2_, 300-μm parasagittal slices were cut with a vibrating microtome (Leica). Slices were incubated in oxygenated artificial cerebrospinal fluid at 32°C for 1.5 h and then transferred to a recording chamber with a planar multielectrode recording setup (MED64, Alpha Med Science, Tokyo, Japan), which was perfused constantly with oxygenated artificial cerebrospinal fluid maintained at 32°C. To induction of LTP, the DG area was stimulated by single monopolar pulses at 30 s intervals and the fEPSPs were recorded in the CA3 area. The input–output signal was recorded and the stimulation intensity corresponding to 30% of the maximal slope was chosen for the test pulses and LTP induction protocol. After fEPSP being stable (the range of variation in amplitude is less than 10% of the average), four consecutive trains (1 s) of stimuli at 100 Hz were applied and the fEPSP recording lasted for 90 min after the stimulus.

The transverse slices of brain (300-μm thick) containing the (DG and CA3) were cut on a vibrating microtome (Leica VT 1000s, Heidelberger, Nussloch, Germany) at 4°C. Slices were continuously infused with oxygen-containing ACSF [consisting of (in mmol/L) 124 NaCl, 25 NaHCO_3_, 2.5 KCl, 1 NaH_2_PO_4_, 2 CaCl_2_, 1 MgSO_4_, and 10 glucose] at room temperature at a rate of 2.5–5 mL/min in a recording chamber, and the experimental operation was performed. The recording pipettes (3–5 MΩ) were filled with a solution containing 120 mmol/L CH_4_SO_3_, 20 mmol/L CsCl, 4 mmol/L NaCl 10 mmol/L HEPES, 0.05 mmol/L EGTA, 4 mmol/L Mg2ATP, 0.2 mmol/L Na3GTP, 5 mmol/L QX-314 (adjusted to pH 7.2 with KOH, 290 mOsmol). The recordings were performed in voltage-clamp mode using an Axon 200B amplififier (Molecular Devices). Clampex and Clampfifit 10.2 softwares (Molecular Devices) were used to acquire and analyse the data. Miniature excitatory postsynaptic currents (mEPSCs) were recorded at holding potentials of −70 mV in the presence of 1 μmol/L tetrodotoxin and 10 μmol/L bicuculline. Miniature postsynaptic currents were measured automatically by setting an appropriate threshold of − 6 pA (2.5 times the SD of the noise). The initial access resistance was 20–40 MΩ and was monitored throughout the experiment. Data were discarded if the access resistance changed >20% during the experiment. Data were filtered at 2 kHz and digitized at 10 kHz.

### Stereotaxic injection and drug administration

Mice were anaesthetized by a mixture of ketamine (100 mg/kg, intraperitoneal) and dexmedetomidine (0.5 mg/kg, intraperitoneal). Holes were drilled above the DG area of the hippocampus (anterior/posterior = −2.0 mm, medial/lateral = ±2.0 mm, dorsal/ventral = −2.2 mm). Agomirs mixture (0.5 μL 200 μmol/L miR-218 agomir and 0.5 μL 100 μmol/L miR-124 agomir), AAVs were microinfused into the hippocampus via a Hamilton microsyringe (Reno, NV), as described before (Deng et al., 2020). The DG area of hippocampus in developmental mice was Converted based on the distance from the anterior to the posterior fontanelle. The behaviour was performed after 1 week for miRNA agomir or 2 weeks for AAVs. Senktide (0.4 mg/kg, *s.c.*, HY-P0187, MedChemExpress), YF-2 (20 mg/kg, HY-16531, *i.p.,* MedChemExpress), and SAHA (50 mg/kg, HY-10221, *i.p.,* MedChemExpress) were delivered by pipette to mice once per day during 7-day social isolation. The drugs were list in TableS8.

### Co-immunoprecipitation, coomassie blue staining, and mass spectrum

Mice were anaesthetized by a mixture of ketamine (100 mg/kg, intraperitoneal) and dexmedetomidine (0.5 mg/kg, intraperitoneal), then mice were decapitated and brains were immediately immersed into ice-cold PBS, and hippocampal tissues were taken and lysed in RIPA buffer (Beyotime, P0013D). After quantification by using the BCA protein assay kit (ThermoFisher, #23225), 1 mg of total extracted protein was incubated with 2 μg of antibodies at 4°C overnight. Normal rabbit IgG was used as a negative control. Then, the mixtures were incubated with protein A + G agarose beads (Beyotime, P2012) for 4 h, washed at least four times with PBS, and were boiled for 10 min in SDS sample buffer (Bio-Rad, #161-0737). Tissue lysates were used as input control. The protein samples were separated by 10% SDS-PAGE gel and transferred to nitrocellulose membranes (Merck Millipore, Burlington, MA, USA). For co-immunoprecipitation, the nitrocellulose membranes were incubated with a primary antibody at 4°C overnight and incubated with a secondary antibody at room temperature for 1 h. For mass spectrum, the membrane was immersed into Coomassie blue staining (0.25% R250 (BioFroxx, #1912GR025), 50% ethanol, 10% acetic acid, and 40% water) for 2–4 h until the gel was dyed a uniform blue. After washing the membrane with a destaining solution (40% ethanol, 10% acetic acid, and 50% water), The distinct protein bands of the RTN3-immunoprecipitation group were cut for mass spectrum as described before (Lin et al., 2020).

### Fluorescence *in situ* hybridization

FISH was performed according to the manufacture’s protocol provided by BOSTER Biological Technology (MK1033) with modifications as described before (Ma et al., 2020). Briefly, 30-μm frozen slices containing dorsal hippocampus region were chosen for FISH. After fixation in 4% paraformaldehyde slices were permeabilized in 1× PBS with 0.5% Triton X-100 (sigma) and RNase Remover V3.0 (Huayueyang, China) for 30 min at 4°C. Slices were blocked by adding pre-hybridization buffer at 37°C for 2–4 h, after that, hybridization was carried out with the specific miRNA FISH probes in a moist chamber at 37°C away from light overnight. The probes were list in TableS8. Slices were washed three times with wash buffer (2× SSC, 0.5× SSC, and 0.2× SSC) at 37°C away from light for 15 min. After the last rinse, slices were incubated with DAPI in the dark room for 10 min. The miRNA probes were designed and synthesized by Tsingke Biotechnology (Beijing, China). All images were obtained by confocal microscope (ZEISS, LSM 800).

### RNA extraction and qRT-PCR

Total RNA was extracted by TRIzol reagent according to the manufacture’s instruction as described before (Bao et al., 2020; De-Yi Liu, 2022). One microgram of total RNA was synthesized cDNA of mRNA by Reverse Transcription kit (Toyobo life science) and cDNA of miRNA by miRcute First strand cDNA Synthesis kit (TIANGEN, Beijing, China). The qRT-PCR was performed with Hieff qPCR SYBR Green Master Mix (Yeasen, cat#11201ES60) on CFX96 Real-Time PCR Detection System (Bio-Rad). The primers were purchased from Tsingke Biological Technology (Beijing, China) and the sequences are detailed in Table S9.

### Prediction of protein–protein binding sites and molecular docking

Proteins structures were collected from the AlphaFold Protein Structure Database (AlphaFold Protein Structure Database (ebi.ac.uk)) (Varadi et al., 2022) and prediction of protein–protein binding sites were performed by using three online prediction tools (HDOCK (HDOCK Server (hust.edu.cn)) and interEvDock3 (InterEvDock3 (univ-paris-diderot.fr))). The amino acids of RTN3 bound by different proteins were recorded and the high frequent amino acids were collected as pocket for molecular docking. The small molecular compounds from Approved drugs in major juridications (ZINC, (docking.org) (Irwin et al., 2020)) were used to perform molecular docking with RTN3 using Discovery studio 2016. After molecular docking, the top 200 poses were chosen for further biological experiments. The structure of protein–protein interaction and protein-small molecular compounds were shown by PyMOL.

### Immunofluorescence

Mice were anaesthetized by a mixture of ketamine (100 mg/kg, intraperitoneal) and dexmedetomidine (0.5 mg/kg, intraperitoneal) and immediately perfused with normal saline and 4% paraformaldehyde solution continuously. The brains were dissected and postfixed for 24 h at 4°C. After fixation, the brains were dehydrated by 30% sucrose in PBS for twice until the brains sank in the solution. 30-μm slices were prepared by using a freezing microtome (Leica). The slices were permeabilized in 1× PBS with 0.5% Triton X-100 (Sigma) for immunofluorescence or in 1× PBS with 0.5% Triton X-100-0.3% H_2_0_2_ for immunohistochemistry (Huang et al., 2023) for 30 min at room temperature and blocked by 3% BSA (Sigma) in PBS for 30 min at room temperature. After that, slices were incubated with primary antibodies for 24 h. The antibodies were list in TableS8. Then, the slices were washed three times and incubated with secondary antibodies (ThermoFisher) for 1 h away from light at 37°C and incubated with DAPI for 10 min. The images were captured under a confocal microscope (ZEISS, LSM 800) as described before (Xie et al., 2019).

### Nuclei extraction and CUT&Tag

Nuclei were extracted from hippocampus tissues according to the manufacture’s protocol provided by Solarbio (Beijing, China) with nuclei extraction kit (SN0020). CUT&Tag was performed according to the manufacture’s protocol provided by Vazyme (TD903, Nanjing, Jiangsu, China) with modifications as described before (Kaya-Okur et al., 2019). Briefly, after nuclei extraction the nuclei were washed twice with Wash Buffer. The nuclei were added into the activated Concanavalin A-coated magnetic beads (ConA Beads) with Binding Buffer and incubated at room temperature for 10 min. After collecting the mixtures of nuclei and ConA Beads, 50 μL precooling Antibody Buffer and H3K4me3 antibody or H3K27ac antibody were added and co-incubated overnight at 4°C. The unbound primary antibody was removed by placing the tube on the magnet stand to clear and pulling off all of the liquid. The secondary antibody diluted 1:50 in 50 μL of Dig-Wash buffer was added and incubated on a rotator at RT for 30 min. Nuclei were washed with the magnet stand three times for 5 min in 200 μL Dig-Wash buffer to remove unbound antibodies. A 100 μL of 1:50 dilution of pA/G-Tnp in Dig-300 buffer was prepared and added to the nuclei with gentle pipetting, which was incubated on a rotator at RT for 1 h. Nuclei were washed 3× for 5 min in 200 μL Dig-300 buffer to remove unbound pA/G-Tnp protein. Next, nuclei were resuspended in 50 μL 1:4 dilution of TTBL in Dig-300 Buffer and incubated at 37°C for 60 min for fragmentation. To stop fragmentation, 5 μL proteinase K, 100 μL buffer L/B, and 20 μL DNA extract beads were added with vortexing and incubated at 55°C for 10 min, and then at 70°C for 20 min to inactivate Proteinase K. The sample was washed with the magnet stand for 2 min in 200 μL Buffer WA and 2 × 2 min in 200 μL Buffer WB. After allowing to dry ~5 min, 22 μL sterile water was added with pipetting, and then the sample was quickly spun and allowed to sit for 5 min. Sample was placed on a magnet stand and the liquid was collected to a fresh tude.

To amplify libraries, 15 μL DNA was mixed with 10 μL of a universal N5XX and N7XX primer. A 25 μL of 2× CAM (CUT&Tag Amplification Mix) was added and mixed. The sample was placed in a thermocycler with a heated lid using the following cycling conditions: 72°C for 3 min; 95°C for 3 min; 15 cycles of 98°C for 10 s; and 60°C for 5 s; final extension at 72°C for 1 min and hold at 4°C. The PCR product was cleaned-up using VAHTS DNA Clean Beads. Paired-end Illumina sequencing was performed on the bar-coded libraries using an Illumina HiSeq 2500 following the manufacturer’s instructions and obtained a 6- to 7-G raw base data. Fastp version 0.23.0 was used to remove adapter and low-quality reads. Align paired-end reads were performed using Hista2 v 2.2.0.

### 3D reconstruction

The analysis of MFBs and mitochondria and synaptic vesicles in MFBs was performed previously with modification (Shi et al., 2022). The virus expressing EGFP (AAV2/8-Syn-EGFP, 1 × 10^12^ IU/μL, 1 μL) was injected into the DG region of hippocampus. After 4 weeks, the mice were isolated and/or administrated by senktide for 7 days. The slices of hippocampus from these mice were obtained and immunostained. The images were obtained by LSM800 confocal microscope (Zeiss) with a 63× oil immersion objective lens and analysed by IMARIS software (Bitplane). We can refactor the whole image or segment only a region of interest. First, to fit into the object, we reconstruct the MFBs using the following customized settings: surface detail 0.3 μm (smooth); diameter of a largest sphere: 3.0; threshold (background subtraction): 25. After surface reconstruction, the filter function was used to wipe off miscellaneous points (volume < 0.05 μm^3^ and area < 0.1 μm^2^). Then, we remodel the filopodia with Filament Tracer module. To evaluate the number of the mitochondria and synaptic vesicles, the ‘mask all’ function was used to mask out the channels that overlap with MFBs. After that, the new channels were reconstructed with the Spots function. Finally, the parameters of surface and spots were exported to separate tables for analysis.

### PP2A activity assay

Mice hippocampus tissue extracts were prepared. PP2A activity in the supernatants by centrifuge was tested using the Serine/Threonine Phosphatase Assay kit (V2460, Promega, Madison, USA) according to the manufacturer’s protocol. Briefly, the high concentration of phosphate in these supernatants is eliminated prior to performing the assay using the supplied Spin Columns, and then the supernatants were normalized according to the protein concentration, 5 μg total protein in triplicates were incubated with a chemically synthesized phosphopeptide (RRA (pT) VA), an optimal substrate for PP2A, PP2B, and PP2C, but not for PP-1 because of its more stringent structural requirements. The buffer for PP2A (PP2A reaction buffer without cation) was added for the incubation for 30 min at 33°C. Phosphate level was detected by measuring the absorbance of molybdate:malachite green:phosphate complex at 630 nm. The PP2A level was calculated according to the standard curve and the PP2A activity was evaluated by PP2A level/5 μg protein/30 min.

### Statistics

GraphPad Prism 8.0 was used to perform all statistical analyses. Data are presented as mean ± SEM unless otherwise stated. A Kolmogorov–Smirnov test or Shapiro–Wilk normality test was used to test the normality. Parametric data were analysed by *t*-test, one-way, or two-way ANOVA followed by Dunnett’s, or Tukey’s post hoc analysis for comparisons of multiple samples. Non-parametric data were analysed by the Mann–Whitney test followed by Dunn’s post hoc analysis for comparisons of multiple samples (TableS10). *P* values < 0.05 were considered statistically significant.

## Supplementary data

Supplementary data is available at *Protein & Cell Journal* online. https://doi.org/10.1093/procel/pwad056.

pwad056_suppl_Supplementary_Figures_1-11_Tables_S1-S2-S7-S9

pwad056_suppl_Supplementary_Tables_S3

pwad056_suppl_Supplementary_Tables_S4

pwad056_suppl_Supplementary_Tables_S5

pwad056_suppl_Supplementary_Tables_S6

pwad056_suppl_Supplementary_Tables_S10

## Data Availability

All data associated with this study are presented in the paper or the Supplementary Materials. The software used in the current study has been cited in ‘Materials and Methods’ section. Please address all requests for reagents and materials to L.Q.Z. (zhulq@mail.hust.edu.cn). All data needed to evaluate the conclusions in the paper are present in the paper and/or Supplementary Materials.

## Code availability

This paper does not report original code.

## Study approval

All of the animal procedures were followed by guidelines and were approved by the Animal Care and Use Committee of Tongji Medical College under approval number 2020-S2317.
