# Supplementary material for: Senktide blocks aberrant RTN3 interactome to retard memory decline and tau pathology in social isolated Alzheimer’s disease mice
Source: Protein Cell. 2023 Nov 27;15(4):261–84. doi: 10.1093/procel/pwad056 (PMC10984625; doi:10.1093/procel/pwad056)
Supplement: pwad056_suppl_Supplementary_Figures_1-11_Tables_S1-S2-S7-S9 [file pwad056_suppl_supplementary_figures_1-11_tables_s1-s2-s7-s9.pdf]

Supplementary Fig. 1. The behavioral changes in isolated AD mice and group housed AD mice at different ages.

(A-C) The open field test was used to detect the anxious-like behavior in different groups. The total distance (A), velocity (B) and time in the center (C) were evaluated.
(D, E) The elevated plus maze task was used to detect the anxious-like behavior in different groups. The time in the open arms (D) and the total entries to the open arm (E) were evaluated.
(F, G) The latencies to reach the hidden platform in the learning stage (day1-day 7) of Morris Water Maze in group-housed and socially isolated WT (F) and AD (G) mice.
(H-J) Latency to the platform region at day 9 (H) the total crossing numbers in the platform region at day 9 (I) and the total time spent in the target quadrant at day 9 (J) of Morris water maze in different groups.
(K-M) The percent of freezing time at day 3 (acquisition stage, K) and day 5 (generalization stage, L and M) of pattern separation task.
(N, O) The percent of freezing time in context C at day 3 (N) and the percent of freezing time in context C and D at day 5 (O) in one-month group-housing mice.
All results are mean ± SEM.

Supplementary Fig. 2. The alterations of adult neurogenesis in the DG region of different groups

(A) The representative images of doublecortin (DCX, white) and nucleus (DAPI, blue) in different groups.
(B) The quantitative analysis for the DCX positive cells in the DG of different groups.
** p<0.01, versus group-housed AD mice. All results are mean ± SEM.

Supplementary Fig. 3. The alterations of DG-CA3 circuit in the different groups

(A) Experimental protocol for MFBs analysis. Mice were injected with human Synapsin Ⅰ promoter-driven AAV2/8- EGFP viruses. After 4 weeks, mice were housed in isolation (1 day, 7 days or 14 days) or in group of 4 mice.

(B, C) The normalized density of the surface area (B) and volume (C) of MFBs (surface area or volume of single MFB/thickness of ROI) in the different groups. The values of the surface area and the volume were list in the bottom of curve by different colors as indicated.

(D, E) The quantitative analysis for normalized surface area (D) and volume (E) of MFBs in the different groups.

(F, G) The cumulative curves for normalized surface area (F) and volume (G) of MFBs after arranging the surface area or volume from large to small in the different groups. The dash lines in different colors indicated the percentage of the number of MFBs when the cumulative surface area or volume reaches to 80% of total surface area or volume.

(H, I) The quantitative analysis for the percentage of the number of MFBs indicated in (F) and (G). ** $p<0.01$, *** $p<0.001$, **** $p<0.0001$.

(J) The average amplitude of Mf mEPSCs in different groups.

(K, L) The average frequency (K) and amplitude (L) of mEPSCs (<45 pA) in different groups. * $p<0.05$.

(M, N) The Golgi staining for the dendritic spines in the CA3 pyramidal neurons. (M) The representative images for spines. (N) The quantification of mushroom spine density. **** $p<0.0001$.

All results are mean ± SEM.

Supplementary Fig. 4. Overexpressing of miR-218 and miR-124 can rescues mEPSC frequencies impaired by social isolation

(A) The RNA quality was validated by the PCR for 28S and 18S RNA. M, RNA marker.

(**B**, **C**) The relative expression levels of miR-218 (B) and miR-124 (C) in group-housed or isolated WT and AD mice. *$p< 0.05$, ***$p< 0.001$.

(D) The relative expression levels of miR-218 and miR-124 in group-housed or isolated AD mice co-treated by miR-218 agomirs and miR-124 agomirs. ****$p< 0.0001$.

(E) The representative mEPSC traces in the different groups.

(F) The amplitude of mEPSC in different groups. ****$p< 0.0001$.

(G) The frequency of mEPSC in different groups. ****$p< 0.0001$.

(H) The representative mEPSC traces and the illustration for the MF based synaptic current. ****$p< 0.0001$.

(I, J) The representative images of doublecortin (DCX, green) and nucleus (DAPI, blue) in different groups (I) and the quantitative analysis (J) for the DCX positive cells in the DG of isolated AD mice co-treated by miR-dDiAs agomirs. ** $p<0.01$, versus scramble treated isolated AD mice.

(K) The expression profiles of precursors for the miR-dDiAs in the DG of in 7-day socially isolated (SI) and group-housed (GH) WT and AD mice. **$p< 0.01$, ***$p< 0.001$, ****$p< 0.0001$.

(L, M) The enhancer regions of miR-dDiAs as indicated by the H3K27ac, H3K4me1 and H3K4me3 peaks.

Supplementary Fig. 5. miR-dDiAs regulate the expression of Rtn3

(A) The protein level of RTN3 was analyzed by western blot. The representative blots (upper) and the quantitative analysis. *$p< 0.05$, ***$p< 0.001$.

(B) The potential miRNAs binding sites in 3'UTR of *Rtn3* (upper) and the detailed sequence of wild type and mutant 3'UTR of *Rtn3* containing the binding sites of miR-218 and miR-124.

(C) The luciferase experiments were used to evaluate the direct binding of miR-218 and miR-124 with the 3'UTR of *Rtn3*. *$p<0.05$, vs scramble treated.

(D, E) The protein level of RTN3 for Figure 4I in the cells treated with miR-218 and miR-124 antagomirs (D) or with miR-218 and miR-124 mimics (E). *$p< 0.05$, ***$p< 0.001$.

(F) The relative expression levels of miR-218, miR-124 and *Rtn3* mRNA upon the treatment of miR-218 antagomirs, miR-124 antagomirs and scrambled control. ***$p< 0.001$, concentration of treatment of antagomir: miR-218, 200 nM; miR-124, 100nM. ****$p< 0.0001$.

(G) The relative expression levels of miR-218, miR-124 and *Rtn3* mRNA upon the treatment of miR-218 mimics, miR-124 mimics and scrambled control. concentration of treatment of mimics: miR-218, 200 nM; miR-124, 100nM. ****$p< 0.0001$.

All results are mean ± SEM.

Supplementary Fig. 6. Knockdown of RTN3 in isolated *Rtn3*$^{\text{flox/+}}$ -AD mice rescues impaired mEPSCs

(A) Upper: Schematic diagram of the *Rtn3*$^{\text{flox/flox}}$ allele and the allele cut by Cre-mediated recombination. Lower: The representative genotyping blots for

Rtn3$^{flox/flox}$ mice. WT: wild type mice; f/f: *Rtn3*$^{flox/flox}$ mice; f/+: *Rtn3*$^{flox/+}$ mice; Neg: negative control; M: marker.
(B, C) The images of body size in different mouse strains (B) and quantification analysis of body weight (C).
(D, E) The representative images of the whole brain in different mouse strains (D) and quantification analysis of brain weight (E).
(F-H) The mRNA (F) and protein (G, H) level of the RTN3 in the hippocampus of different mouse strains. * p<0.05, ** p<0.01, *** p< 0.001, **** p< 0.0001.
(I) The representative mEPSC traces in the different groups.
(J) The frequency of mEPSC in different groups. ****p< 0.0001.
(K) The amplitude of mEPSC in different groups. ****p< 0.0001.
(L) The representative mEPSC traces and the illustration for the MF based synaptic current. ****p< 0.0001.
(M) The representative images of doublecortin (DCX, green) and nucleus (DAPI, blue) and the quantitative analysis for the DCX positive cells in the DG of isolated Rtn3 cKO AD mice.

All results are mean ± SEM.

Supplementary Fig. 7. Upregulation of RTN3 induced by miR-dDiAs decoy was rescues by *Rtn3* shRNA.

(A) Schematic representation of the miR decoy. Ten miR-218 and five miR-124 target sequences were inserted into the 3’ UTR of a GFP reporter gene driven by human synapsin 1 promoter. Poly(A), polyadenylation tail. Dashes indicate that there is no nucleotide at that position in the sequence.
(B) The diagram for the virus injection in WT mice and the representative fluorescent image.
(C-E) The level of miRNAs and *Rtn3* mRNA (C) and protein (D, E) level of the RTN3 in the hippocampus. * p<0.05, *** p< 0.001, **** p< 0.0001.
(F, G) The percent of freezing time at day 3 (acquisition stage, F) and day 5 (generalization stage, G) of pattern separation task.

Supplementary Fig. 8. RTN3 binds with mitochondria associated proteins, vesicle associated proteins and PP2A B subunits.

(A) The DG region of hippocampus from 7-day socially isolated and group-housed AD mice was co-immunoprecipitated by RTN3 antibody and subjected to SDS-PAGE gels followed by Coomassie blue staining analysis. Three bands were selected for

further analysis by mass spectrometry.

(B-E) Identification of RTN3 binding protein RAB3B (B), SLC25A46 (C), DNM1L (D) and SYNGR1 (E) by mass spectrometry. aa: amino acid.

(F) The GO analysis for the proteins that enriched in RTN3 interactome of social isolated mice (fold change ≥ 1.5) by mass spectrum. The color of dots indicates the different adjust *p* value and the size of dots indicates the different numbers of identified proteins.

(G) The gene set enrichment analysis for the proteins that enriched in RTN3 interactome of social isolated mice (fold change ≥ 1.5) by Cytoscape. The size of dots indicates the number of gene size.

Supplementary Fig. 9. RTN3 induced hyperphosphorylation of Tau through binding with PP2A B subunits, but did not affect the phosphorylation of GSK-3β.

(A) PPP2R5A co-immunoprecipitated with PPP2CA and Tau5 in hippocampus.

(B) PPP2R2C co-immunoprecipitated with PPP2CA and Tau5 in hippocampus.

(C) PPP2R5E co-immunoprecipitated with PPP2CA and Tau5 in hippocampus.

(D) PP2A activity assay. **** $p<0.0001$.

(E) Quantification analysis of the phosphorylation of tau at Ser396 (pSer396) for Figure 5N. *$p< 0.05$.

(F) Analysis of the phosphorylation of tau at Thr231 (pT231), Ser262 (pS262), Ser202 and Thr205 (AT8) in cells transfected with P301L-GFP and *Rtn3*.

(G, H) The protein level of GSK3β and the phosphorylation of GSK3β at Ser9 was analyzed by western blot. The representative blots (g) and the quantitative analysis (h).

(I) Quantification analysis of His for Figure 5P. **$p< 0.01$.

All results are mean ± SEM.

Supplementary Fig. 10. Senktide disrupts the binding between RTN3 and its partners.

(A) The wide-type and mutated *Rtn3* mRNA and protein sequence used to analyze the key amino acids that is required for binding to RTN3 partners. *Rtn3* WT: *Rtn3* wild type, *Rtn3* Mut: the indicated amino acids of RTN3 were mutated to alanine (A) or leucine (L).

(B-E) The HEK293 cells were transfected with pcDNA3.1(+)-*Rtn3*-WT-His or pcDNA3.1(+)-*Rtn3*-Mut-His plus pcDNA3.1(+)-*Rab3b*-Flag (B), pcDNA3.1(+)-*Syngr1*-Flag (C), pcDNA3.1(+)-*Dnm1l*-Flag (D), pcDNA3.1(+)-*Slc25a46*-Flag (E) plasmids, separately. After 48 hours, the cell lysis was collected and 5% volume of cell lysates were used for input, half volume of the remaining cell lysis was coimmunoprecipitated with anti-IgG or anti-His separately and then subjected to the western blot by using anti-Flag.

(F-I) Hydrogen bond donor/acceptor of binding site in RTN3 with Carbetocin (blue, F), Glycerol Phenylbutyrate (blue, G), Saralasin (blue, H), Aripiprazole Lauroxil (blue, I). RTN3 is labeled with green while the amino acids interacting with indicated drugs were labeled with magenta. The green dotted lines indicated hydrogen bond between the compound and RTN3.

(J-M) The HEK293 cells were transfected with pcDNA3.1(+)-*Rtn3*-His plus the pcDNA3.1(+)-*Rab3b*-Flag (J), pcDNA3.1(+)-*Dnm1l*-Flag (K), pcDNA3.1(+)-*Slc25a46*-Flag (L) and pcDNA3.1(+)-*Syngr1*-Flag (M)plasmids and then treated with the indicated drugs for 48 hours. Then, the cell lysis was collected and immunoprecipitated with anti-His and then subjected to the western blot by using anti-Flag.

(N) Scheme for treatment of social isolated AD mice with senktide. Senktide was administrated to the 2 months isolated AD mice during the 7 days isolation with the dose of 0.4mg/kg by *s.c.* injection every day.

Supplementary Fig. 11. Senktide inhibits the binding between Rtn3 and its partners in vivo.

(A, B) Rtn3 co-immunoprecipitated with PPP2R2C, PPP2R5A and PPP2R5E in hippocampus.

(C, D) PPP2R5A co-immunoprecipitated with PPP2CA and Tau5 in hippocampus.

(E, F) PPP2R2C co-immunoprecipitated with PPP2CA and Tau5 in hippocampus.
(G, H) PPP2R5E co-immunoprecipitated with PPP2CA and Tau5 in hippocampus.
(I, J) RTN3 co-immunoprecipitated with DNM1L, RAB3B, SLC25A46 and SYNGR1 in hippocampus.
(K) The immunofluorescence of pS396 in the stratum lucidum of CA3 region.

Supplementary Table 1 patient information

Number	Age	Sex	Final diagnosis
1	85	M	Multiple organ failure
2	90	M	Liver cancer
3	84	M	Respiratory failure
4	84	M	Multiple organ failure
5	87	M	Multiple organ failure
6	85	M	AD
7	88	M	AD
8	95	M	AD
9	81	M	AD

Supplementary Table 2 list of top 40 DG enriched miRNAs

miRNA name	Ct value
mmu-miR-194	26.90
mmu-miR-187	27.74
mmu-miR-27a	27.28
mmu-miR-27b	27.39
mmu-let-7a	21.80
mmu-let-7d	26.43
mmu-miR-128	29.13
mmu-let-7c	22.96
mmu-miR-99b	18.22
mmu-miR-361	24.52
mmu-miR-151	24.92
mmu-miR-181a	19.83
mmu-miR-132	25.93
mmu-miR-127	26.97
mmu-miR-26a	23.94
mmu-miR-191	17.48
mmu-miR-125a	26.18
mmu-miR-125b	23.29

mmu-miR-24	23.70
mmu-let-7b	16.54
mmu-miR-342	22.47
mmu-let-7e	25.93
mmu-miR-103	23.38
mmu-miR-221	21.92
mmu-miR-222	19.64
mmu-miR-107	30.92
mmu-miR-139	21.82
mmu-miR-23a	25.01
mmu-miR-23b	29.41
mmu-miR-16	25.11
mmu-miR-138	21.89
mmu-miR-15b	26.76
mmu-miR-29a	19.13
mmu-miR-335	24.01
mmu-miR-539	28.44
mmu-miR-15a	28.52
mmu-miR-185	26.34
mmu-miR-210	26.25
mmu-miR-124	30.46
mmu-miR-218	18.78

Supplementary Table 7. The predicted compounds that docked to the Rtn3 pocket

zinc_id	Libdock score	Molecule Names	Cat NO.
ZINC000150340074	236.347		
ZINC000095615286	222.768	Senktide	HY-P0187
ZINC000195761836	219.775		
ZINC000169289386	216.689	Saralasin	HY-P0205
ZINC000169368439	214.577		
ZINC000150340074	212.726		
ZINC000261106254	210.867		
ZINC000169368439	209.293		
ZINC000095617677	208.834		
ZINC000169368439	208.506		
ZINC000072131413	208.409	hydroxy ritonavir	
ZINC000261106252	206.951		
ZINC000095615286	206.59	Senktide	HY-P0187
ZINC000095615286	204.971		
ZINC000410428644	204.527		
ZINC000169368439	203.397		
ZINC000150338506	201.065	inositol niacinate	
ZINC000169289386	200.677	Saralasin	HY-P0205

ZINC000169289386	199.427	Saralasin	HY-P0205
ZINC000085427689	199.095		
ZINC000410428644	198.891		
ZINC000085537068	198.243	pralmorelin	
ZINC000169368439	197.945		
ZINC000150340074	197.562		
ZINC000150338703	197.404	Carbetocin	HY-17573
ZINC000085537068	197.31	pralmorelin	
ZINC000169368439	197.224		
ZINC000085537068	196.842	pralmorelin	
ZINC000150338506	196.629	inositol niacinate	
ZINC000008214644	196.339	pentagastrin	
ZINC000095564895	195.52	Aripiprazole lauroxil	HY-108751
ZINC000169289386	195.15	Saralasin	HY-P0205
ZINC000195761836	194.839		
ZINC000095617677	194.077		
ZINC000169289386	193.911	Saralasin	HY-P0205
ZINC000085574641	193.719		
ZINC000169289386	193.706	Saralasin	HY-P0205
ZINC000085537068	192.612	pralmorelin	
ZINC000169368439	192.465		
ZINC000169368439	191.83		
ZINC000169289386	191.386	Saralasin	HY-P0205
ZINC000085537068	191.103	pralmorelin	
ZINC000261106252	190.497		
ZINC000195761836	190.315		
ZINC000085537068	190.165	pralmorelin	
ZINC000169289386	190.057	Saralasin	HY-P0205
ZINC000072131413	189.393	hydroxy ritonavir	
ZINC000049918329	189.129		
ZINC000008214644	188.903	pentagastrin	
ZINC000169289386	188.35	Saralasin	HY-P0205
ZINC000038945666	189.353	Glycerol phenylbutyrate	HY-B2087

Supplementary Table 8. Antibodies, probes and drugs. Antibodies

Antibody name	Dilution	Cat NO.	Manufacturer
RTN3 Polyclonal antibody	1:200 for IF<br>1:1000 for WB	12055-2-AP	Proteintech
Anti-TIMM44 antibody	1:200 for IF	HPA043052	Sigma-Aldrich
anti-FLAG antibody	1:1000 for WB	20543-1-AP	Proteintech
His-Tag Monoclonal antibody	1:1000 for WB	66005-1-Ig	Proteintech
Synaptogyrin-3 antibody	1:200 for IF	sc-271046	Santa cruz

Beta Actin Polyclonal antibody	1:1000 for WB	20536-1-AP	Proteintech
Doublecortin	1:200 for IF	4604s	Cell Signaling Technology
Anti-Histone H3 (acetyl K27) antibody	1:100 for CUT&Tag	ab4729	Abcam
PPP2CA Polyclonal antibody	1：1000 for WB	13482-1-AP	Proteintech
PPP2R2C Polyclonal antibody	1：1000 for WB	12747-1-AP	Proteintech
PPP2R5A Polyclonal antibody	1：1000 for WB	12675-2-AP	Proteintech
PPP2R5E Polyclonal antibody	1：1000 for WB	23885-1-AP	Proteintech
Dnm1l Polyclonal antibody	1：1000 for WB	12957-1-AP	Proteintech
Anti-Synaptogyrin 1 antibody	1：1000 for WB	ab113886	Abcam
SLC25A46 antibody	1：1000 for WB	sc-515810	Santa cruz
RAB3B Monoclonal Antibody	1：1000 for WB	bsm-51316M	Bioss
Tau5 Monoclonal Antibody	1：1000 for WB	AHB0042	Invitrogen
Phospho-Tau (Ser202, Thr205) Monoclonal Antibody (AT8)	1：1000 for WB	MN1020	Invitrogen
Tau (Phospho-Ser396) Antibody	1：1000 for WB	#11102	Signalway
Solar Fluor 488	1:200 for IF	#S1063	Solarbio

IF, immunofluorescence; WB, Western Blot.

Probes

miRNA	Sequence 5'-3'
miR-124	GGCATTCACCGCGTGCCTTA
miR-218	ACATGGTTAGATCAAGCACAA

Drugs

Drug name	Concentration	Cat NO.	Manufacturer
Senktide	5 μm in vitro<br>0.4 mg/kg *s.c.*	HY-P0187	MedChemExpress
Saralasin	5 μm in vitro	HY-P0205	MedChemExpress
Carbetocin	10 μm in vitro	HY-17573	MedChemExpress

Glycerol phenylbutyrate	10 µm in vitro	HY-B2087	MedChemExpress
Aripiprazole lauroxil	10 µm in vitro	HY-108751	MedChemExpress
YF-2	20 mg/kg *i.p.*	HY-16531	MedChemExpress
SAHA	50 mg/kg *i.p.*	HY-10221	MedChemExpress
miR-218-5p agomir	0.5 µL 200 µM in vivo	miR40000663-4-5	RIBOBIO
miR-218-5p mimic	200 nM in vitro	miR10000663-1-5	RIBOBIO
miR-218-5p antagomir	200 nM in vitro	miR312919151936-4-5	RIBOBIO
miR-124-3p mimic	100 nM in vitro	miR10000134-1-5	RIBOBIO
miR-124-3p agomir	0.5 µL 100 µM in vivo	miR40000134-4-5	RIBOBIO
miR-124-3p antagomir	100 nM in vitro	miR30000134-4-5	RIBOBIO

Plasmids and viruses

pEGFP-C1	Clontech	6084-1
pEGFP-C1- P301L	This lab	NA
pCDNA3.1(+)-*Rtn3*-6×His	This paper	NA
pCDNA3.1(+)-*Dnm1l*-3×FLAG	This paper	NA
pCDNA3.1(+)-*Syngr1*-3×FLAG	This paper	NA
pCDNA3.1(+)-*Rab3b*-3×FLAG	This paper	NA
pCDNA3.1(+)-*Slc25a46*-3×FLAG	This paper	NA
pCDNA3.1(+)-*Rtn3*-3*A/L-6×His	This paper	NA
pCDNA3.1(+)-*Rtn3*-5*A -6×His	This paper	NA
pCDNA3.1(+)-*Rtn3*-6*A -6×His	This paper	NA
pCDNA3.1(+)-*Rtn3*-7*A -6×His	This paper	NA
pCDNA3.1(+)-*Rtn3*-17*A -6×His	This paper	NA
psiCheck2.0-*Rtn3* 3'UTR	This paper	NA
psiCheck2.0-*Rtn3* Mut1	This paper	NA
psiCheck2.0-*Rtn3* Mut2	This paper	NA
psiCheck2.0-*Rtn3* Mut3	This paper	NA
AAV-hSYN-miR-218 & 124 decoys	Obio	Custom made
AAV-Hsyn-*Rtn3* shRNA	Obio	Custom made

Supplementary Table 9. primers used for qPCR and plasmid construction

qPCR primer	5'-3'
Rtn3 F	CTGGCAGCCTTCAGTGTTATC
Rtn3 R	ATCGCAGCATTCATGTAGTTG
Sirt2 F	CCTGATATCGTGTTTTTCGG
Sirt2 R	CTTGTTAATGAGCAGCCGTG
Hdac1 F	GCACCAAGAGGAAAGTCTGT
Hdac1 R	TATTATCTGGGCGAATAGAA

Hdac7 F	CAGGATCGGCTCAAACCTCA
Hdac7 R	GGCCATCATTCGCCATAGGT
Hdac8 F	ACGGGAAGTGTAAAGTAGCC
Hdac8 R	TCGATGTAAAACTGAAGGCA
Sirt6 F	GAATGCTCGGCCCTCGAAGA
Sirt6 R	GGTGCCCACAACCGTGTCTC
Hdac3 F	AGAGAGTGGCCGCTACTATT
Hdac3 R	TTCCCCATGTCCTCGAATGC
Hdac6 F	CCAGCCTCGCATACAAACAA
Hdac6 R	AAGTCAGACACACCCAGTTC
Sirt1 F	ACACCTCTTCATATTTCGGA
Sirt1 R	TTCTTGTGGTTTTTCTTCCA
Hdac10 F	GTCAGATAAGGAAGGAAAAC
Hdac10 R	ATGTAGATGAGGCAAAGGTT
Hdac4 F	AGTTCTCACTGCCCTTGGAA
Hdac4 R	GGGAGCTGTGCTGTGTCTTC
Sirt3 F	GGCACTACAGGCCCAATGTC
Sirt3 R	GCTGCTCCCCAAAGAACACA
Sirt5 F	CCAGTTGTGTTGTAGACGAA
Sirt5 R	AGTTTTAAATAAGGTTCCGT
Hdac11 F	CCGGTCATCTTTCTTCCCAA
Hdac11 R	AGTCTCGCTCATGCCCATTG
Hdac5 F	CGCTACGACAACGGGAACTT
Hdac5 R	CTGGGCTTTTGCTGCAAGAC
Hdac9 F	AGCCCATCTCACCTTTAGAC
Hdac9 R	GCTTGCCACTGCCCTTTCTC
Sirt7 F	CTTTGGGGAGAGGGGGACAT
Sirt7 R	GTTGGTGGGAGCGGTTGTAG
EP300 F	AATGGGGAAGTGAGGCAGTG
EP300 R	TGGGGTTGTGGTGGAATCTG
Crebbp F	AACCAAAACGACTACAGGAG
Crebbp R	TGAATCACAAAGAATACCTC
Sirt4 F	ACTCCTCGTGATGACAGGCG
Sirt4 R	CCCACAAAGTTTCGGGCCCA
Hdac2 F	CAGTTGCCCTTGATTGTGAA
Hdac2 R	CTCCTTTGGGGTCTGTTTTC
Hat1 F	ATCTTGAGAATGACATTAGA
Hat1 R	GAACAGTGTTGACAGGCTAC
miR-218-5p	TTGTGCTTGATCTAACCATGT
miR-124-3p	TAAGGCACGCGGTGAATGCC
miR-210-3p	CTGTGCGTGTGACAGCGGCTGA
miR-185-5p	TGGAGAGAAAGGCAGTTCCTGA
miR-15a-5p	TAGCAGCACATAATGGTTTGTG
miR-539-5p	GGAGAAATTATCCTTGGTGTGT

miR-335-3p	TTTTTCATTATTGCTCCTGACC
miR-29a-3p	TAGCACCATCTGAAATCGGTTA
miR-15b-5p	TAGCAGCACATCATGGTTTACA
miR-138-5p	AGCTGGTGTTGTGAATCAGGCCG
miR-16-5p	TAGCAGCACGTAAATATTGGCG
miR-23b-3p	ATCACATTGCCAGGGATTACC
miR-23a-3p	ATCACATTGCCAGGGATTTCC
miR-139-5p	TCTACAGTGCACGTGTCTCCAG
miR-107-3p	AGCAGCATTGTACAGGGCTATCA
miR-222-3p	AGCTACATCTGGCTACTGGGTCT
miR-221-3p	AGCTACATTGTCTGCTGGGTTTC
miR-103-3p	AGCAGCATTGTACAGGGCTATGA
let-7e-5p	TGAGGTAGGAGGTTGTATAGTT
miR-342-3p	TCTCACACAGAAATCGCACCCGT
let-7b-5p	TGAGGTAGTAGGTTGTGTGGTT
miR-24-3p	TGGCTCAGTTCAGCAGGAACAG
miR-125b-5p	TCCCTGAGACCCTAACTTGTGA
miR-125a-5p	TCCCTGAGACCCTTTAACCTGTGA
miR-191-5p	CAACGGAATCCCAAAAGCAGCTG
miR-26a-5p	TTCAAGTAATCCAGGATAGGCT
miR-127-3p	TCGGATCCGTCTGAGCTTGGCT
miR-132-5p	AACCGTGGCTTTCGATTGTTAC
miR-181a-5p	AACATTCAACGCTGTCGGTGAGT
miR-151-3p	CTAGACTGAGGCTCCTTGAGG
miR-361-3p	TCCCCCAGGTGTGATTCTGATTTGT
miR-99b-5p	CACCCGTAGAACCGACCTTGCG
let-7c-5p	TGAGGTAGTAGGTTGTATGGTT
miR-128-3p	TCACAGTGAACCGGTCTCTTT
let-7d-5p	AGAGGTAGTAGGTTGCATAGTT
let-7a-5p	TGAGGTAGTAGGTTGTATAGTT
miR-27a-3p	TTCACAGTGGCTAAGTTCCGC
miR-187-3p	TCGTGTCTTGTGTTGCAGCCGG
miR-194-5p	TGTAACAGCAACTCCATGTGGA
pre-miR-218 F	TGCTTGCGAGGTATGAGAAA
pre-miR-218 R	TAGAAAGCTGCGTGACGTTC
pre-miR-124 F	CTCTCTCTCCGTGTTCACAG
pre-miR-124 R	GCCTTAATTGTATGGACATT
Slit2 F	CTTCGGGTAGATGCTTTTCA
Slit2 R	AATCCGCTAGCCACTTGAGA
Slit3 F	AATAGGATCAAGGAAGTGCG
Slit3 R	TTGTCATAGAGAGAGACAGCAG
miR124-2hg F	ACAAACCGAAGGACCTGACCA
miR124-2hg R	GGCTCTTTTCACAGCATCCCTT
Mir124a-1hg F	GATTGGAGAGATCAACGCTG

Mir124a-1hg R	GACAATGAGATAACAGCCACGT
Universal R Primer	GCTGTCAACGATACGCTACG
U6 for	GATGACACGCAAATTCGTGAA
Gapdh for	TGTTTCCTCGTCCCGTAG
Gapdh rev	CAATCTCCACTTTGCCACT
hsa-miR-124	TAAGGCACGCGGTGAATGCC
hsa-miR-218	TTGTGCTTGATCTAACCATGT

primer for plasmid construction	5'-3'
Rtn3 F	TGAGTCAGTCAGTCTGTCGGA
Rtn3 R	TCCTTCATAGTACAAGTGATGATG
Slc25a46 F	GTGACTTCCGGTTGTCAGTCT
Slc25a46 R	AACACAGTGACCTGAATCCAAG
Rab3b F	CTGCCTCTCACCCACTATCG
Rab3b R	GGGAATGGACAGTAATGGAGA
Syngr1 F	ACGATGGAAGGGGGTGCGTA
Syngr1 R	GTGGCAGAGCAGCAGAGGAAG
Dnm1l F	CGGAGGAGAAGAGGAAGCAAG
Dnm1l R	AGGCAGCAGCAGGTTCAAGTC
Rtn3 F for miR-218	GCATCAGTTACTAAAACACCATT
Rtn3 R for miR-218	GATTCAATCTTTATTCTTTACGG
Rtn3 F for miR-124	AAATAGTATGGGGCAAGAGTG
Rtn3 R for miR-124	GCATTCTGGGAGTTCTGTAAG
Rtn3 F-1 for miR-124	TTTCAAGGTATTTGATGGTCAC
Rtn3 R-1 for miR-124	TGTTAGGAGACCCCATAGACC
Rtn3-3*A/L R	AGCGCATAGTGCAGCAGCCCCGCCGCCGCCGCCGAGAGCT
Rtn3-3*A/L F	TGCTGCACTATGCGCTGCCCTGGGGGCGAAGAGCTGC
Rtn3 5*A R-1	GAGCCGCATGCCTTCGCCCCCAGGGCGGGGCAGGCT
Rtn3 5*A F-1	GAAGGCATGCGGCTCCTCGTGTGCGGTGCACGATCT
Rtn3 5*A R-2	TGCCACATCTCGAGCGAAAATCAGATCGTGCACCGCA
Rtn3 5*A F-2	GCTCGAGATGTGGCAAAGACTGGGTTTGTCTTTGGCA
Rtn3 6*A R-1	TGCGGCAAAGACAGCCCCAGTCTTCTTCACATCTC
Rtn3 6*A F-1	GCTGTCTTTGCCGCAACACTGATCATGCTGCTCTC
Rtn3 6*A R-2	CAGCATTGCCAGTGTGGTGCCAAAGACA
Rtn3 6*A F-2	ACACTGGCAATGCTGCTCTCTCTGGCAGC
Rtn3 7*A R	AGCGGCTGCCAGAGCAGCCAGCATGATCAGTGTGGTGC
Rtn3 7*A F	GCTGCTCTGGCAGCCGCTAGTGTTATCAGTGTGGTCTC
Rtn3 17*A R	TCAGAGCCATAGCGACAGCCAGCTTCAAGGAGT
Rtn3 17*A F	TCGCTATGGCTCTGATGACCTATGTTGGTGC

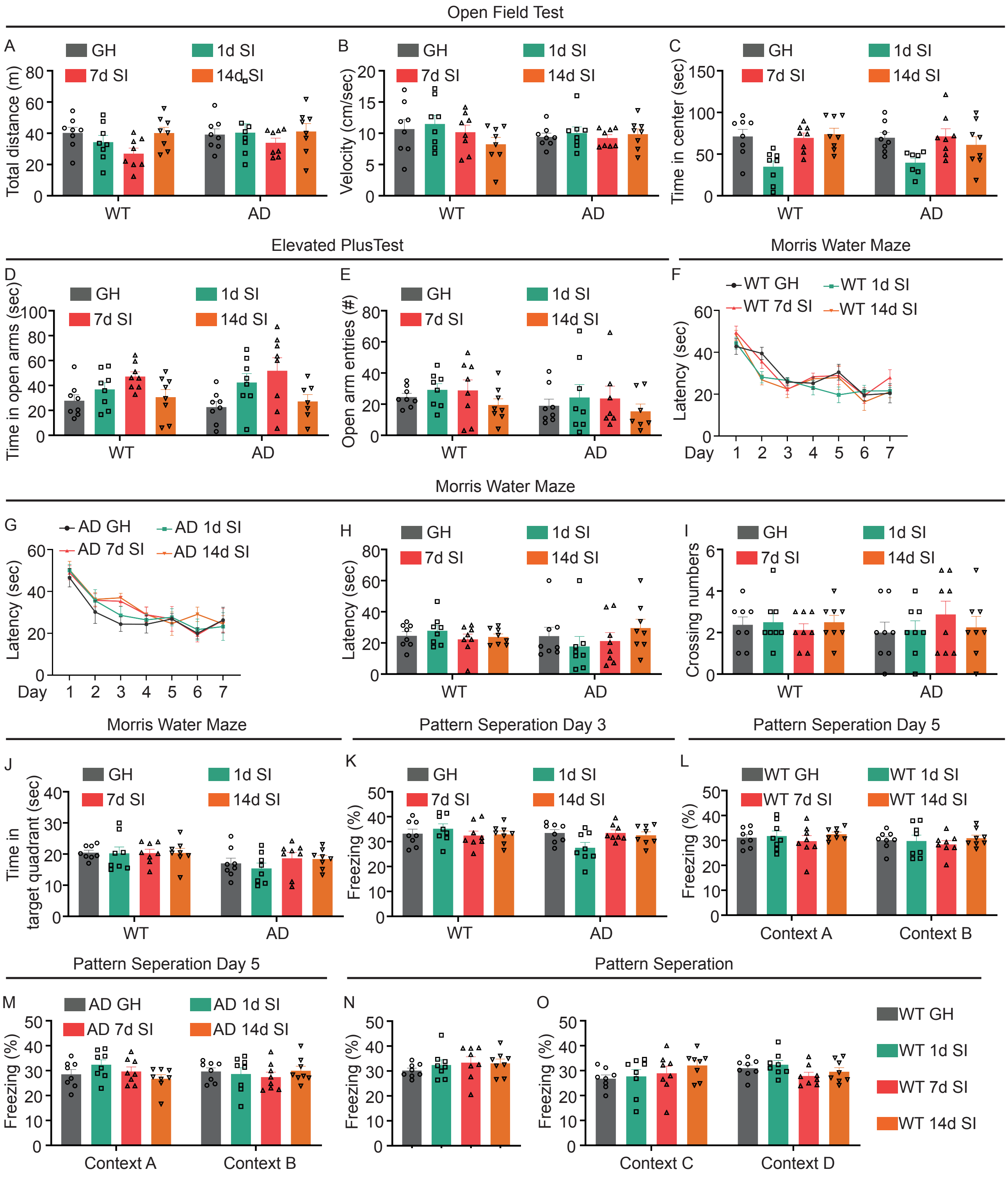

Open Field Test
A
GH
1d SI
7d SI
14d SI
Total distance (m)
WT
AD
B
Velocity (cm/sec)
C
Time in center (sec)
Elevated PlusTest
D
Time in open arms (sec)
E
Open arm entries (#)
Morris Water Maze
F
WT GH
WT 1d SI
WT 7d SI
WT 14d SI
Latency (sec)
Day
Morris Water Maze
G
AD GH
AD 1d SI
AD 7d SI
AD 14d SI
H
I
Crossing numbers
Morris Water Maze
Pattern Seperation Day 3
Pattern Seperation Day 5
J
Time in target quadrant (sec)
K
Freezing (%)
L
Context A
Context B
Pattern Seperation Day 5
Pattern Seperation
M
N
O
Context C
Context D

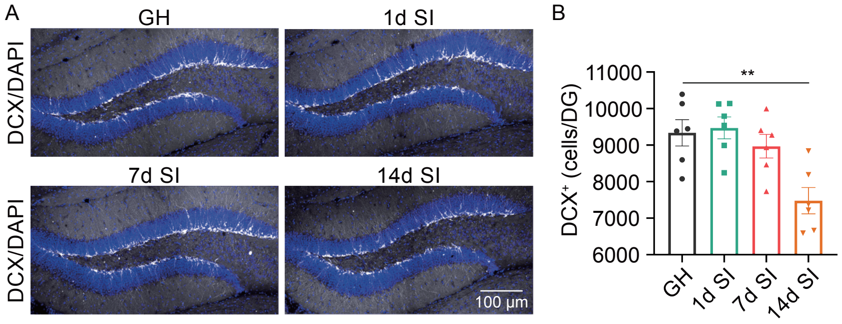
A
GH
1d SI
7d SI
14d SI
DCX/DAPI
DCX/DAPI
100 μm
B
DCX⁺ (cells/DG)
11000
10000
9000
8000
7000
6000
**
GH
1d SI
7d SI
14d SI

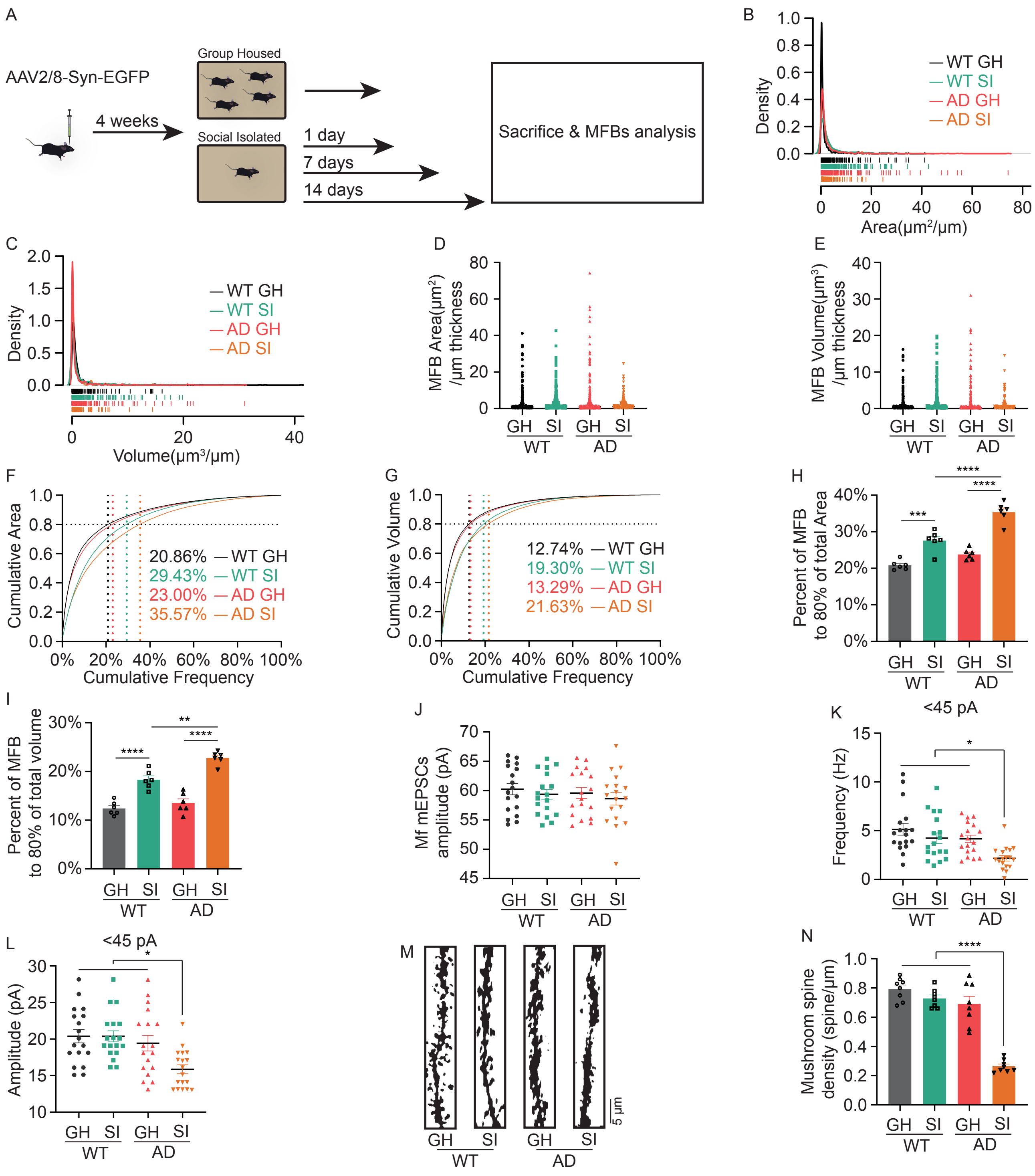

A
AAV2/8-Syn-EGFP
4 weeks
Group Housed
Social Isolated
1 day
7 days
14 days
Sacrifice & MFBs analysis
B
Density
WT GH
WT SI
AD GH
AD SI
Area(μm²/μm)
C
Density
Volume(μm³/μm)
D
MFB Area(μm²) /μm thickness
GH SI GH SI
WT AD
E
MFB Volume(μm³) /μm thickness
F
Cumulative Area
20.86% WT GH
29.43% WT SI
23.00% AD GH
35.57% AD SI
Cumulative Frequency
G
Cumulative Volume
12.74% WT GH
19.30% WT SI
13.29% AD GH
21.63% AD SI
Cumulative Frequency
H
Percent of MFB to 80% of total Area
I
Percent of MFB to 80% of total volume
J
Mf mEPSCs amplitude (pA)
K
<45 pA
Frequency (Hz)
L
<45 pA
Amplitude (pA)
M
5 μm
N
Mushroom spine density (spine/μm)

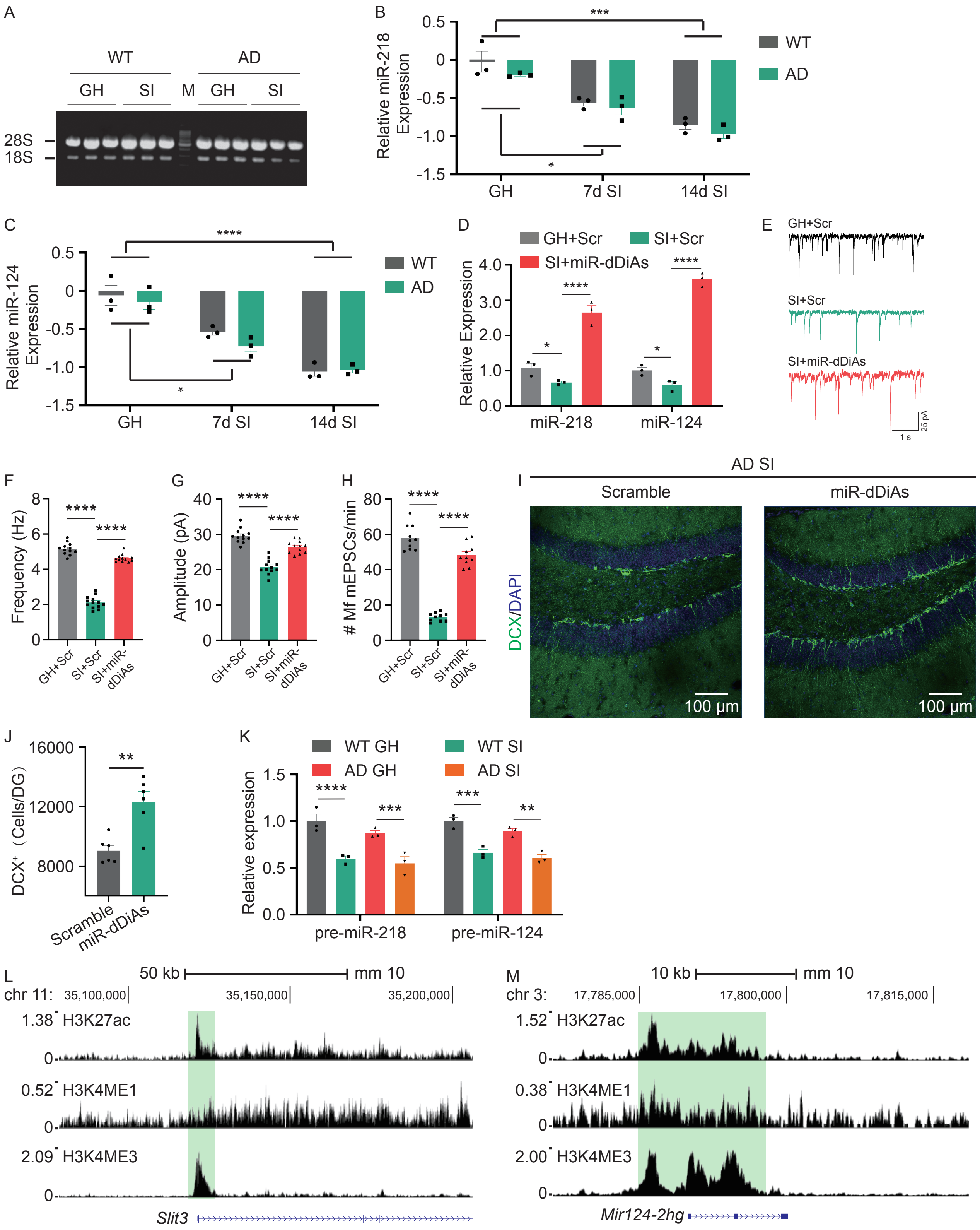
A
WT
AD
GH
SI
M
28S
18S
B
Relative miR-218 Expression
WT
AD
GH
7d SI
14d SI
C
Relative miR-124 Expression
D
GH+Scr
SI+Scr
SI+miR-dDiAs
Relative Expression
miR-218
miR-124
E
GH+Scr
SI+Scr
SI+miR-dDiAs
25 pA
1 s
F
Frequency (Hz)
G
Amplitude (pA)
H
Mf mEPSCs/min
I
AD SI
Scramble
miR-dDiAs
DCX/DAPI
100 μm
J
DCX⁺（Cells/DG)
Scramble
miR-dDiAs
K
WT GH
WT SI
AD GH
AD SI
Relative expression
pre-miR-218
pre-miR-124
L
50 kb
mm 10
chr 11:
35,100,000
35,150,000
35,200,000
H3K27ac
H3K4ME1
H3K4ME3
Slit3
M
10 kb
chr 3:
17,785,000
17,800,000
17,815,000
Mir124-2hg

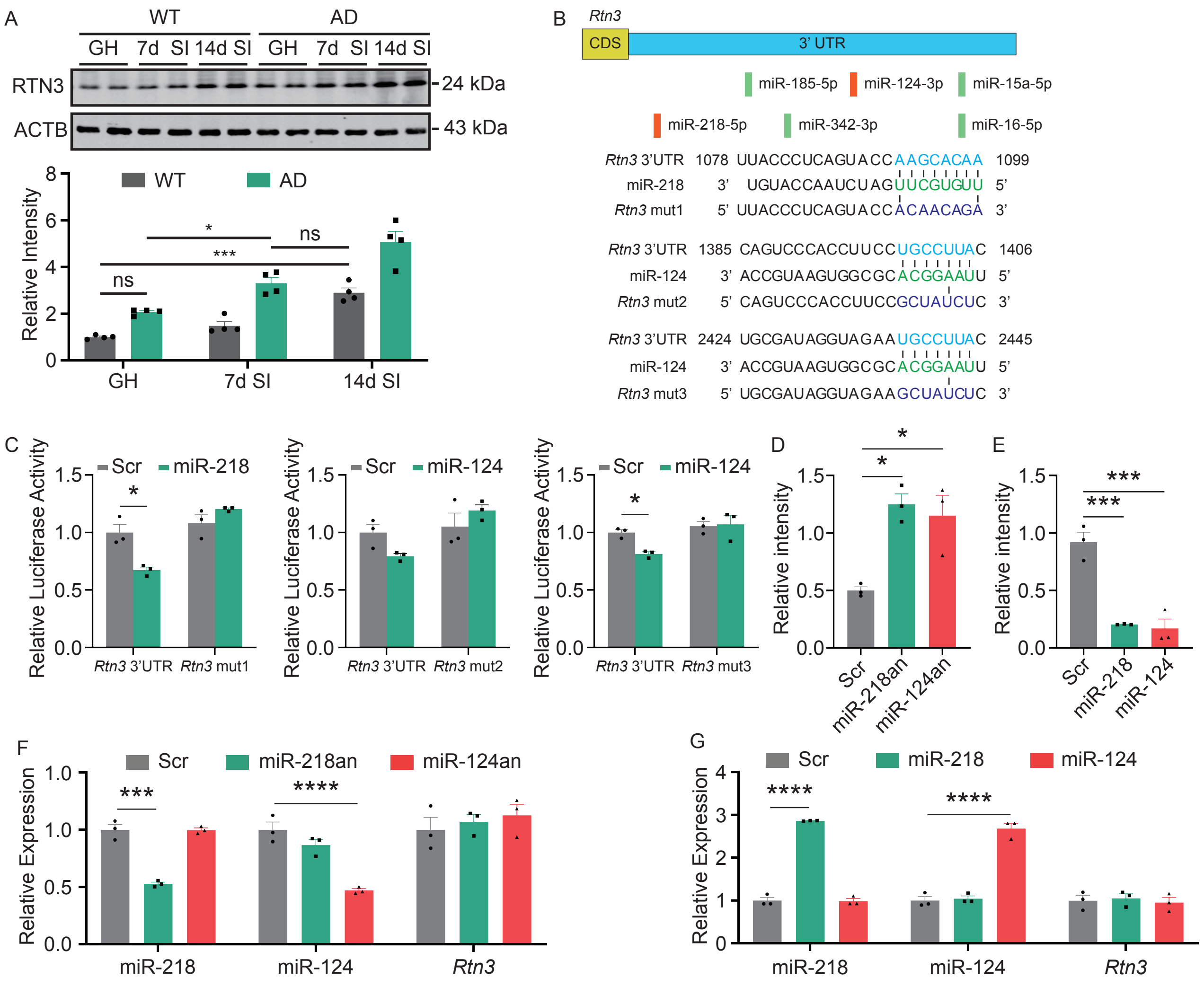
A
WT
AD
GH 7d SI 14d SI GH 7d SI 14d SI
RTN3
24 kDa
ACTB
43 kDa
Relative Intensity
WT AD
ns
*
***
ns
GH
7d SI
14d SI
B
Rtn3
CDS
3' UTR
miR-185-5p miR-124-3p miR-15a-5p
miR-218-5p miR-342-3p miR-16-5p
Rtn3 3'UTR 1078 UUACCCUCAGUACCAAGCACAA 1099
miR-218 3' UGUACCAAUCUAGUUCGUGUU 5'
Rtn3 mut1 5' UUACCCUCAGUACCACAACAGA 3'
Rtn3 3'UTR 1385 CAGUCCCACCUUCCUGCCUUAC 1406
miR-124 3' ACCGUAAGUGGCGCACGGAAUU 5'
Rtn3 mut2 5' CAGUCCCACCUUCCGCUAUCUC 3'
Rtn3 3'UTR 2424 UGCGAUAGGUAGAAUGCCUUAC 2445
miR-124 3' ACCGUAAGUGGCGCACGGAAUU 5'
Rtn3 mut3 5' UGCGAUAGGUAGAAGCUAUCUC 3'
C
Relative Luciferase Activity
Scr miR-218
Rtn3 3'UTR Rtn3 mut1
Scr miR-124
Rtn3 3'UTR Rtn3 mut2
Scr miR-124
Rtn3 3'UTR Rtn3 mut3
D
Relative intensity
Scr
miR-218an
miR-124an
E
Relative intensity
Scr
miR-218
miR-124
F
Relative Expression
Scr miR-218an miR-124an
***
****
miR-218
miR-124
Rtn3
G
Relative Expression
Scr miR-218 miR-124
****
****
miR-218
miR-124
Rtn3

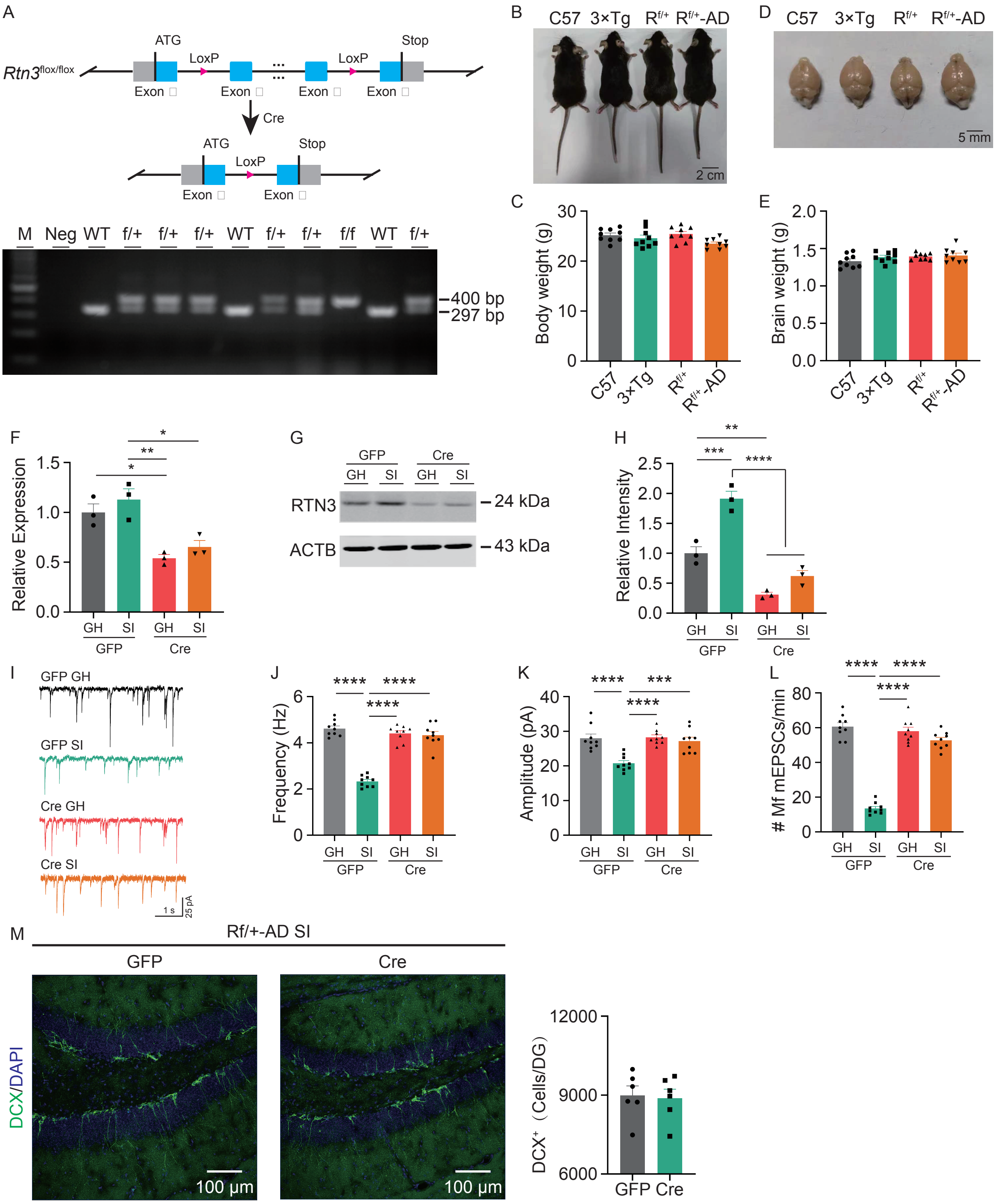
A
Rtn3flox/flox
ATG
LoxP
Stop
Exon
Cre
M Neg WT f/+ f/+ f/+ WT f/+ f/+ f/f WT f/+
400 bp
297 bp
B
C57 3×Tg Rf/+ Rf/+-AD
2 cm
D
5 mm
C
Body weight (g)
E
Brain weight (g)
F
Relative Expression
GH SI GH SI
GFP Cre
G
RTN3
ACTB
24 kDa
43 kDa
H
Relative Intensity
I
GFP GH
GFP SI
Cre GH
Cre SI
1 s
25 pA
J
Frequency (Hz)
K
Amplitude (pA)
L
Mf mEPSCs/min
M
Rf/+-AD SI
GFP
Cre
DCX/DAPI
100 μm
DCX+（Cells/DG)

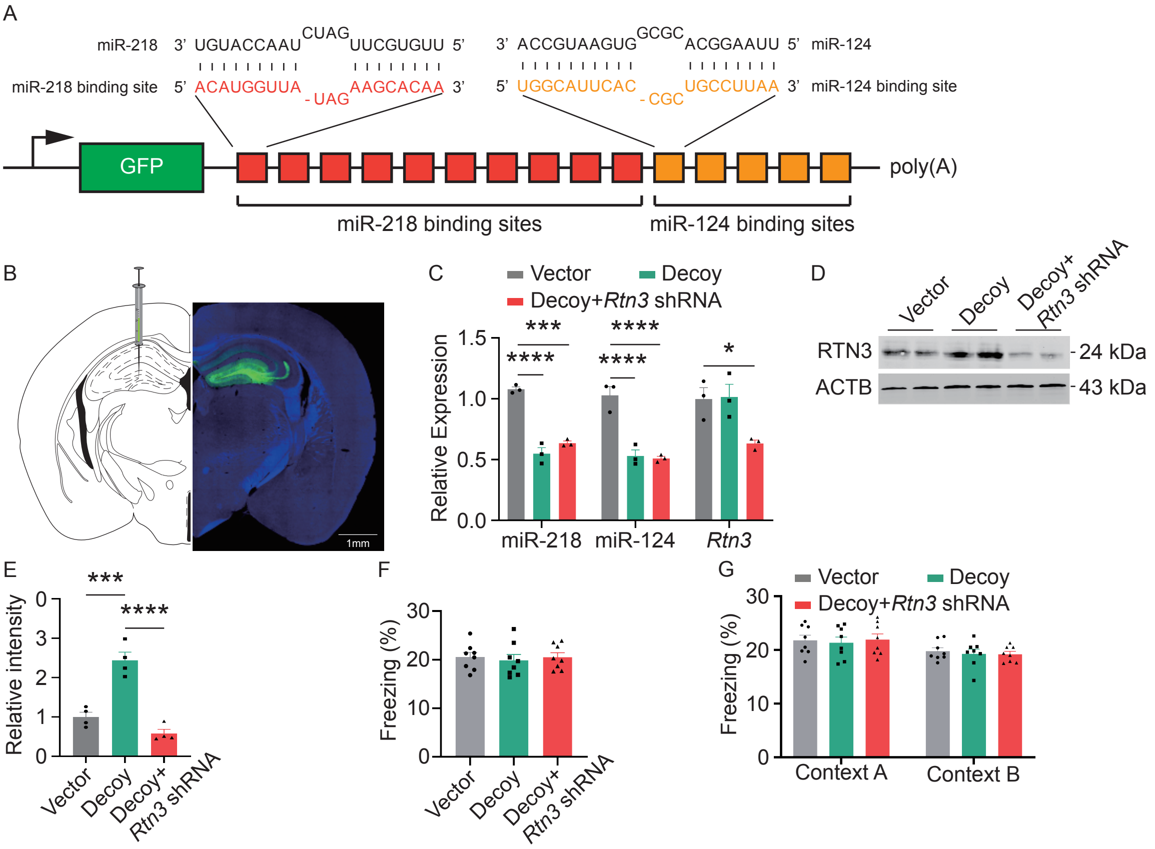
A
miR-218 3' UGUACCAAU CUAG UUCGUGUU 5'
miR-218 binding site 5' ACAUGGUUA - UAG AAGCACAA 3'
3' ACCGUAAGUG GCGC ACGGAAUU 5' miR-124
5' UGGCAUUCAC - CGC UGCCUUAA 3' miR-124 binding site
GFP
poly(A)
miR-218 binding sites
miR-124 binding sites
B
1mm
C
Vector
Decoy
Decoy+Rtn3 shRNA
Relative Expression
miR-218
miR-124
Rtn3
D
Vector
Decoy
Decoy+ Rtn3 shRNA
RTN3
ACTB
24 kDa
43 kDa
E
Relative intensity
Vector
Decoy
Decoy+ Rtn3 shRNA
F
Freezing (%)
Vector
Decoy
Decoy+ Rtn3 shRNA
G
Vector
Decoy
Decoy+Rtn3 shRNA
Freezing (%)
Context A
Context B

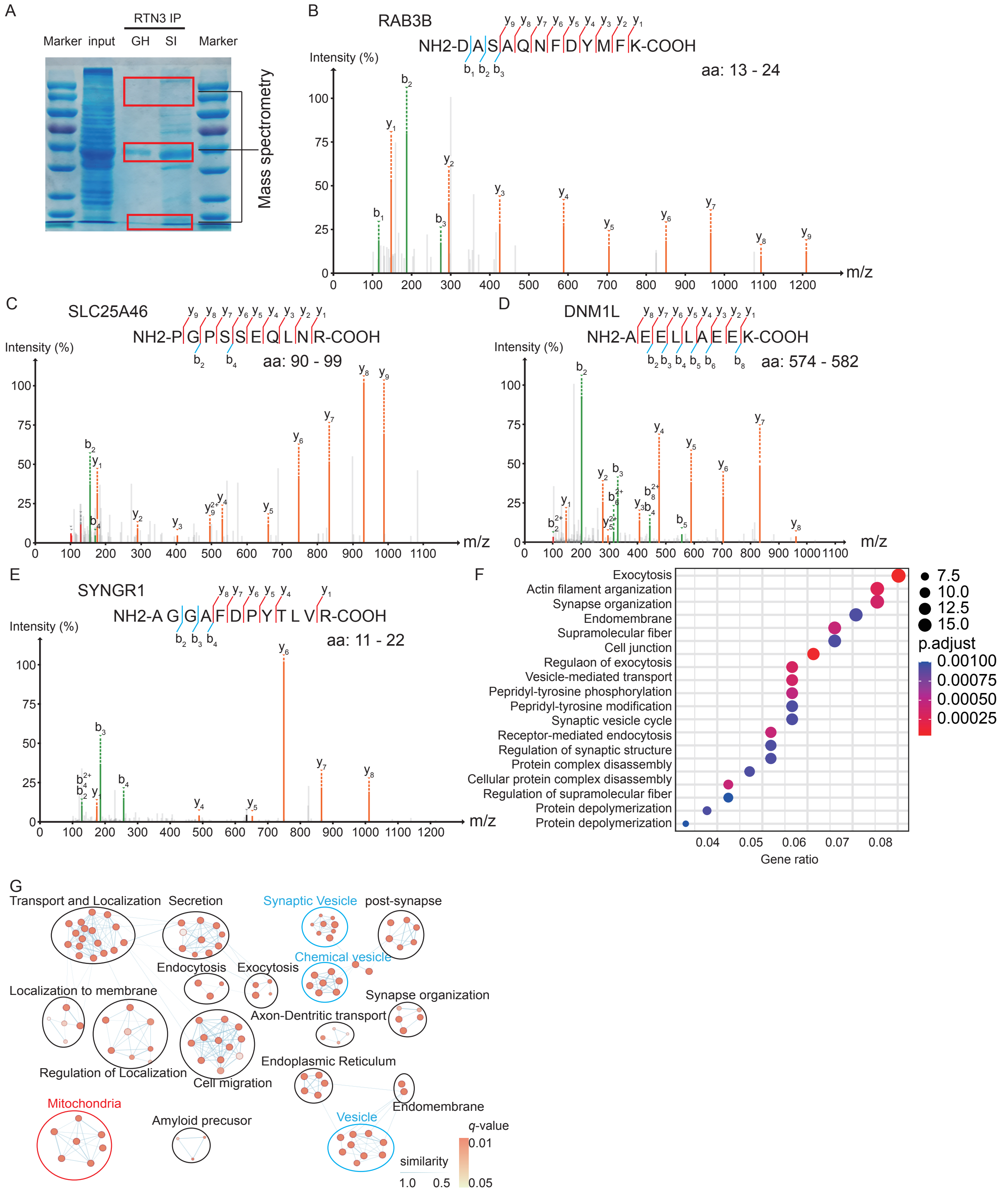

A
RTN3 IP
Marker input GH SI Marker
Mass spectrometry
B
RAB3B
NH2-DASAQNFDYMFK-COOH
aa: 13 - 24
Intensity (%)
m/z
C
SLC25A46
NH2-PGPSSEQLNR-COOH
aa: 90 - 99
Intensity (%)
m/z
D
DNM1L
NH2-AEELLAEEK-COOH
aa: 574 - 582
Intensity (%)
m/z
E
SYNGR1
NH2-AGGAFDPYTLVR-COOH
aa: 11 - 22
Intensity (%)
m/z
F
Exocytosis
Actin filament organization
Synapse organization
Endomembrane
Supramolecular fiber
Cell junction
Regulaon of exocytosis
Vesicle-mediated transport
Pepridyl-tyrosine phosphorylation
Pepridyl-tyrosine modification
Synaptic vesicle cycle
Receptor-mediated endocytosis
Regulation of synaptic structure
Protein complex disassembly
Cellular protein complex disassembly
Regulation of supramolecular fiber
Protein depolymerization
Protein depolymerization
Gene ratio
7.5
10.0
12.5
15.0
p.adjust
0.00100
0.00075
0.00050
0.00025
G
Transport and Localization
Secretion
Synaptic Vesicle
post-synapse
Endocytosis
Exocytosis
Chemical vesicle
Localization to membrane
Synapse organization
Axon-Dentritic transport
Endoplasmic Reticulum
Regulation of Localization
Cell migration
Endomembrane
Mitochondria
Amyloid precusor
Vesicle
q-value
similarity

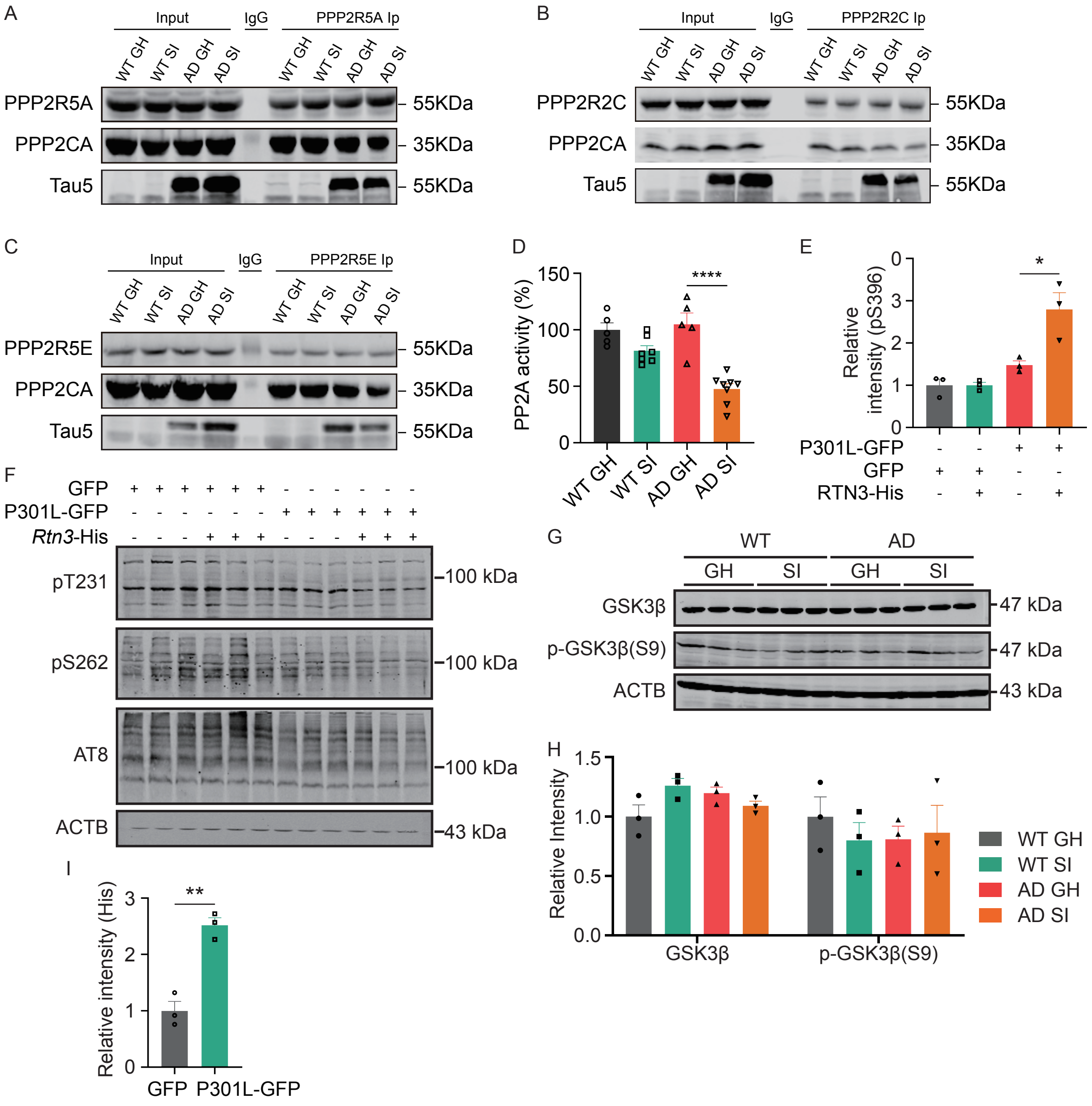

A
Input
IgG
PPP2R5A Ip
WT GH
WT SI
AD GH
AD SI
PPP2R5A
PPP2CA
Tau5
55KDa
35KDa
55KDa
B
PPP2R2C Ip
PPP2R2C
PPP2CA
Tau5
C
PPP2R5E Ip
PPP2R5E
PPP2CA
Tau5
D
PP2A activity (%)
****
E
Relative intensity (pS396)
*
P301L-GFP
GFP
RTN3-His
F
GFP
P301L-GFP
Rtn3-His
pT231
pS262
AT8
ACTB
100 kDa
43 kDa
G
WT
AD
GH
SI
GSK3β
p-GSK3β(S9)
ACTB
47 kDa
43 kDa
H
Relative Intensity
GSK3β
p-GSK3β(S9)
WT GH
WT SI
AD GH
AD SI
I
Relative intensity (His)
**
GFP
P301L-GFP

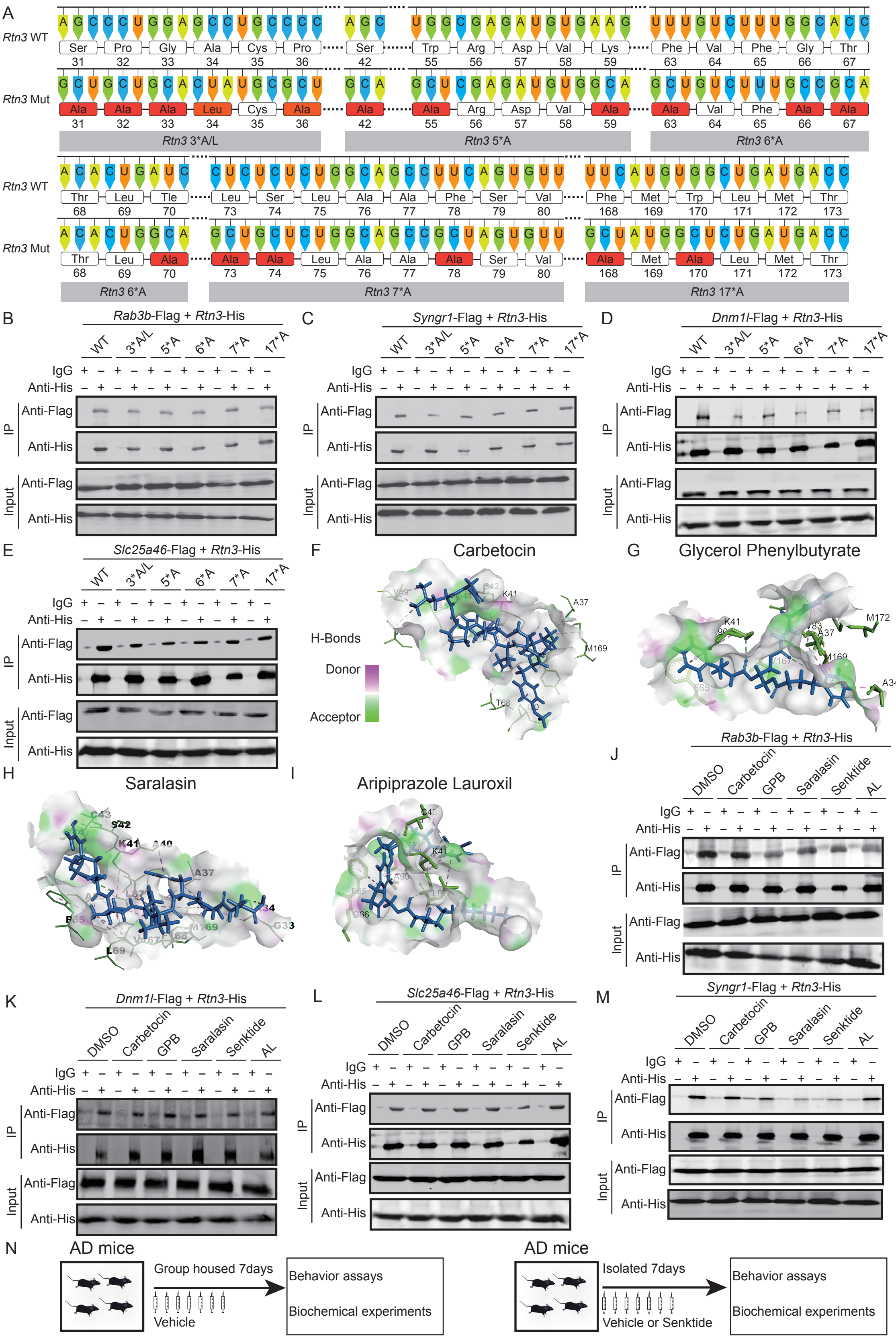
A
Rtn3 WT
Rtn3 Mut
Rtn3 3*A/L
Rtn3 5*A
Rtn3 6*A
Rtn3 7*A
Rtn3 17*A
B
Rab3b-Flag + Rtn3-His
C
Syngr1-Flag + Rtn3-His
D
Dnm1l-Flag + Rtn3-His
E
Slc25a46-Flag + Rtn3-His
WT 3*A/L 5*A 6*A 7*A 17*A
IgG
Anti-His
IP
Input
Anti-Flag
Anti-His
F
Carbetocin
H-Bonds
Donor
Acceptor
G
Glycerol Phenylbutyrate
H
Saralasin
I
Aripiprazole Lauroxil
J
Rab3b-Flag + Rtn3-His
DMSO Carbetocin GPB Saralasin Senktide AL
K
Dnm1l-Flag + Rtn3-His
L
Slc25a46-Flag + Rtn3-His
M
Syngr1-Flag + Rtn3-His
N
AD mice
Group housed 7days
Vehicle
Behavior assays
Biochemical experiments
AD mice
Isolated 7days
Vehicle or Senktide
Behavior assays
Biochemical experiments

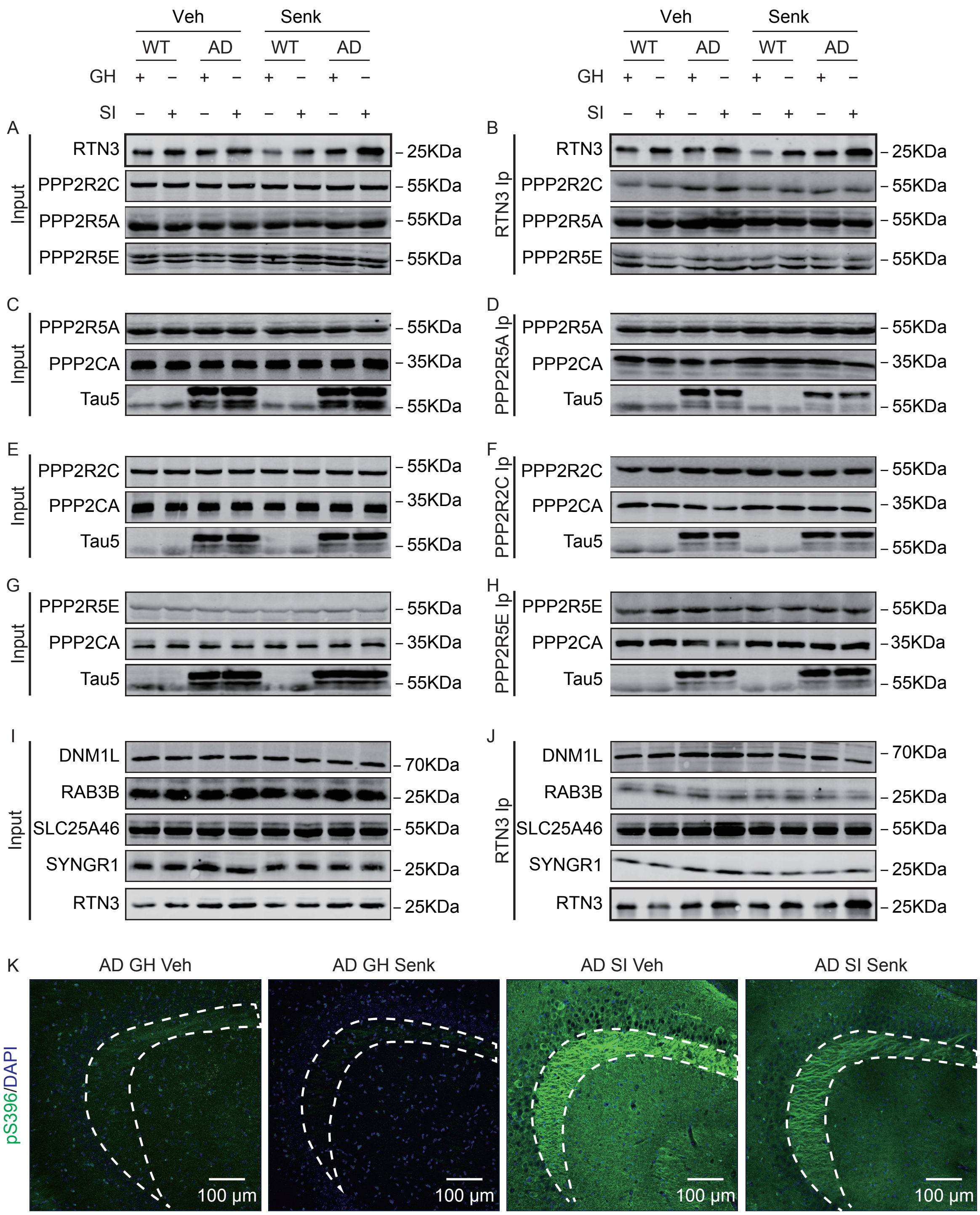

Veh
Senk
WT
AD
GH
SI
A
B
C
D
E
F
G
H
I
J
K
Input
RTN3 Ip
PPP2R5A Ip
PPP2R2C Ip
PPP2R5E Ip
RTN3
PPP2R2C
PPP2R5A
PPP2R5E
PPP2CA
Tau5
DNM1L
RAB3B
SLC25A46
SYNGR1
25KDa
35KDa
55KDa
70KDa
AD GH Veh
AD GH Senk
AD SI Veh
AD SI Senk
pS396/DAPI
100 μm
